# Management of long COVID-19 in children and adolescents: from diagnosis to therapeutically approaches

**DOI:** 10.1080/07853890.2026.2642510

**Published:** 2026-03-11

**Authors:** Olga Adriana Caliman –Sturdza, Roxana Gheorghita, Andrei Lobiuc, Roxana Filip, Iuliana Soldanescu, Serghei Mangul, Mihai Dimian

**Affiliations:** ^a^ Faculty of Medicine and Biological Sciences, Stefan cel Mare University of Suceava, Suceava, Romania; ^b^ Suceava Emergency Clinical County Hospital, Suceava, Romania; ^c^ Integrated Center for Research, Development, and Innovation for Advanced Materials, Nanotechnologies, Manufacturing and Control Distributed Systems (MANSiD), Stefan cel Mare University of Suceava, Suceava, Romania; ^d^ Department of Clinical Pharmacy, USC Alfred E. Mann School of Pharmacy and Pharmaceutical Sciences, University of Southern California, Los Angeles, California, USA; ^e^ Department of Quantitative and Computational Biology, USC Dornsife College of Letters, Arts and Sciences, University of Southern California (USC), Los Angeles, California, USA; ^f^ Department of Computer, Electronics and Automation, University of Suceava, Suceava, Romania

**Keywords:** Long COVID-19, children, adolescents, post-acute sequelae (PASC), diagnosis, rehabilitation, multidisciplinary management, therapeutic interventions

## Abstract

**Introduction:**

Long Coronavirus disease 2019 (COVID-19), also termed post-acute sequelae of severe acute respiratory syndrome coronavirus 2 infection (PASC), has emerged as a complex multisystem condition in children and adolescents worldwide. It can occur even after mild or asymptomatic acute infections, with symptoms that may persist, fluctuate, or relapse over time. This review aims to comprehensively explore the characteristic manifestations, management and current therapeutic possibilities of pediatric Long COVID-19 (L-C19).

**Methods:**

A systematic search was conducted in multiple databases such as PubMed, Scopus, Web of Science, and Google Scholar, for literature published between January 2020 and October 2025.

**Results:**

Diagnosing pediatric L-C19 is challenging due to the heterogeneity of symptoms and lack of specific diagnostic biomarkers. Most young patients experience gradual improvement over months, but a significant subset remains symptomatic for >1 year with substantial disability, underscoring the need for timely diagnosis and intervention. Current clinical consensus emphasizes an individualized, multidisciplinary management approach focused on symptom relief and functional rehabilitation. No definitive cure exists for L-C19; thus, care is tailored to each patient’s predominant issues. Therapeutic strategies combine supportive self-management (e.g. energy conservation and pacing) with both non-pharmacological and pharmacological interventions. Multimodal rehabilitation programs – including graded exercise therapy and cognitive behavioral therapy – have shown promise in improving fatigue, mental health, and overall quality of life. Targeted treatments for specific sequelae (such as autonomic dysfunction or chronic pain) are applied on a case-by-case basis, although high-quality evidence for medications remains limited. Globally, interdisciplinary collaborations have been established to provide harmonized diagnostic and treatment protocols, and major research initiatives are underway to evaluate novel therapies and include children in L-C19 clinical trials.

**Conclusion:**

Ongoing international efforts to develop standardized diagnostic tools, outcome measures, and evidence-based interventions are crucial to optimize care and long-term outcomes for children and adolescents affected by L-C19.

## 1. Introduction

**Figure 1.**
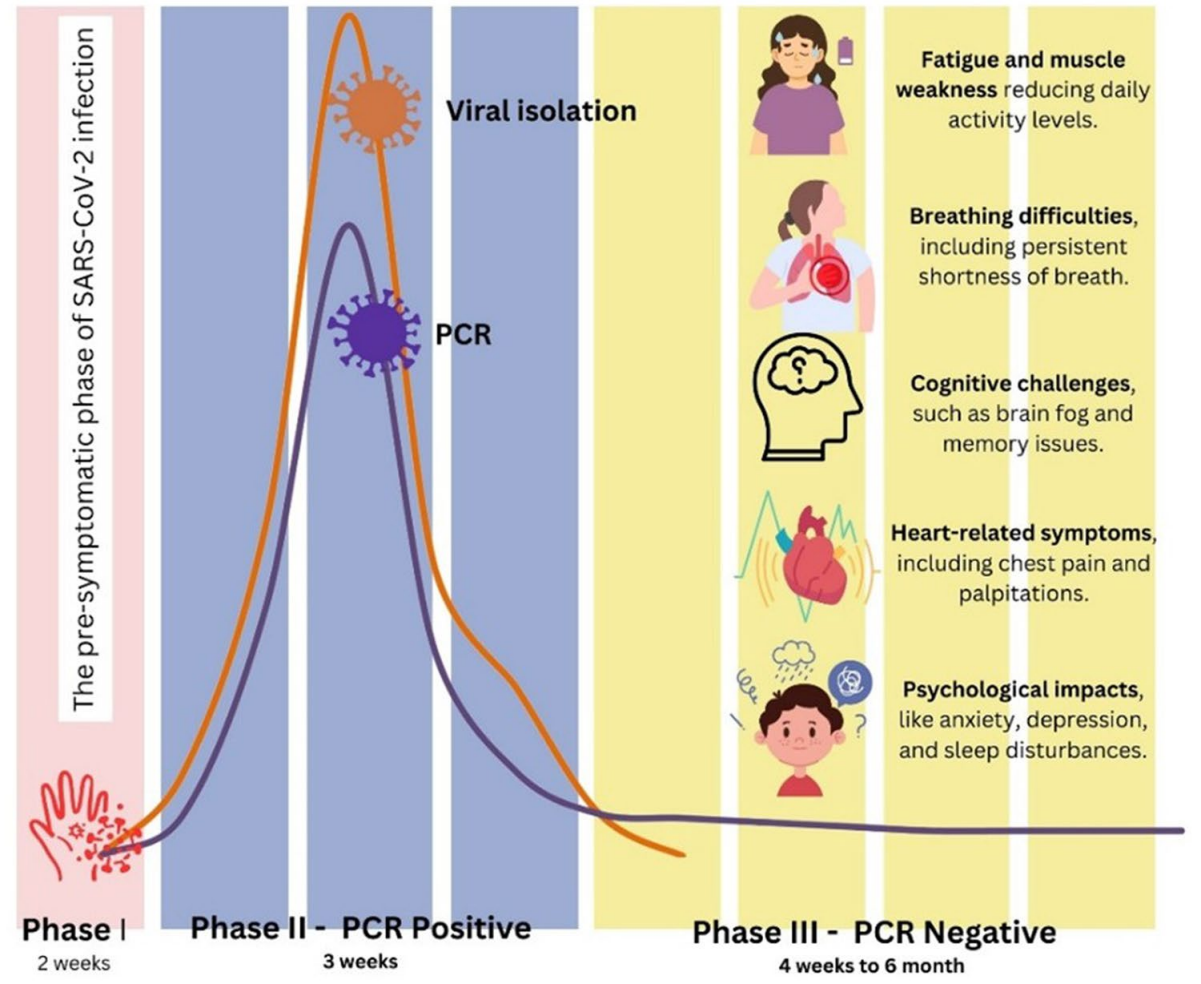
Time course and post-acute complications of L-C19.

Since the start of the COVID-19 pandemic, it has become clear that recovery from acute severe acute respiratory syndrome coronavirus 2 (SARS-CoV-2) infection is not always complete within the expected timeframe. A fraction of the patients have enduring, recurrent, or new symptoms, which may continue to persist months and impact various organ systems [1]. The concept of long COVID (LC-19) was used in the early years of the pandemic through patient communities: it is widely attributed to Dr. Elisa Perego, who used the hashtag *#LongCOVID* on social media in 2020 to describe prolonged illness after suspected/confirmed infection [2]. The term long haulers that describes people with long-term post-infectious symptoms also became popular among other related patient-led and journalistic articles, which contributed to attracting a medical and research interest to the condition [3]. Several names are in the literature, such as long COVID, post-COVID syndrome, post-acute sequelae of SARS-CoV-2 infection (PASC) and post-COVID-19 condition (PCC) [3]. Notably, these entities are defined by major health organizations with various time frames and conceptual frameworks. According to the WHO clinical case definition, post-COVID-19 condition is said to be present in persons with a history of probable or confirmed SARS-CoV-2 infection, usually 3 months from onset, with symptoms persist at least 2 months and they cannot be ascribed to another diagnosis [4,5]. The categories of time-based described in the NICE guideline include ongoing symptomatic COVID-19 (under 4 weeks to 12 weeks) and post-COVID-19 syndrome (symptoms after 12 weeks) not due to an alternative diagnosis [6]. The NIH also has terminology Long COVID/long-haul COVID/PASC to describe an issue of health that may persist or manifest following infection and timing thresholds often start weeks or months following acute illness [7]. Other studies define pediatric post-acute sequelae of SARS-CoV-2 as the persistence of at least one physical symptom for a minimum of 12 weeks after the initial infection with SARS COV-2, such symptoms interfere with daily life, may appear and disappear occasionally, and cannot be attributed to other diseases [8–12] –(Figure 1).

Since imprecise use of terminology may result in conceptual ambiguity and impede cross-study comparisons, the review will employ established terms in WHO and NICE to define pediatric post-acute outcomes and perform an umbrella term when referring to the more general literature, which is in line with the recent synthesis activities that highlight the importance of harmonizing the terminology [9,13]. Children and adolescents tend to have milder acute COVID-19 than adults, but some of them have persistent symptoms, which may influence school attendance, physical activity, cognition, and psychosocial wellbeing [14]. The frequency of pediatric cases is estimated in a wide range across settings since there are differences in defining cases, follow-up time, variants in circulation, comparison groups, and ways of ascertaining the symptoms. The big cohort and longitudinal studies still perfect the prevalence estimates and risk profile among children and adolescents [15,16]. Early pediatric reports included small case series—for example, a Swedish report describing five children (median age of about 12 years) with symptoms persisting for 6–8 months, including fatigue, dyspnea, palpitations/chest pain, and cognitive complaints [17]. Although this type of observation was relevant to hypothesis generation, conclusions about age-specific vulnerability or pattern of symptoms distribution must be viewed with caution when relying on very small samples. Later larger-scale studies have reported a broad spectrum of symptoms among children, often including fatigue, headache, sleeping disturbance, cognitive impairment (so-called brain fog), breathing problems, muscle/joint aches, and palpitations [18]. As an illustration, an early survey of pediatrics in Italy (*n* = 129) found that a significant proportion of children had at least one enduring symptom several months following diagnosis, and functional impact in a significant proportion; this study helped motivate more rigorous pediatric cohort investigations (noting that early versions circulated as a preprint) [19]. Further longitudinal and registry-based research has confirmed the existence of a quantifiable sub-group of children who may suffer from long-term morbidity, which has led to the necessity of pediatric-specific assessment and follow-up routes [20]. Based on their findings, Assady-Pooya et al. considered that L-C19 is a common condition among children and adolescents, and it is essential for the scientific community to delve deeper into the pathophysiology of this condition to develop effective treatments and provide adequate care to affected patients [21].

The diagnosis of pediatric long COVID is complicated by a number of factors. First, persistence of the symptoms should be viewed in the light of the anticipated recovery of the acute infection, which depends on the severity of the infection, as well as host factors [14,19]. Second, not every child has virologic confirmation, particularly at the beginning of the pandemic or in areas with a low access rate to testing, and certain infections are asymptomatic, and it is challenging to attribute them retrospectively. If such individuals later develop multiple symptoms, diagnosing L-C19 becomes problematic without prior evidence of SARS-CoV-2 infection [13]. Residual symptoms in individuals who test negative for COVID-19 also contribute to the diagnostic challenge, as false-negative results can occur if testing is conducted too early or too late in the course of the disease. Furthermore, the immune response to the virus varies; approximately 20% of individuals do not produce detectable antibodies (seroconversion), and antibody levels may decline over time, making it difficult to retrospectively confirm a past SARS-CoV-2 infection [22]. Third, symptoms can be non-specific including fatigue, headache, sleeping disturbance, or problems with concentration, and could be overlapping with other post-infectious syndromes, other mental health stressors, or pandemic-related disruptions [4]. Therefore, WHO and NICE focus on the need to rule out alternative diagnoses and to use functional impact in assessment [23,24]. Lastly, validated pediatric biomarkers remain an unmet requirement and the heterogeneity of study designs still hinders cross-study comparability, reinforcing calls for standardized definitions and carefully designed pediatric cohorts [4].

## 2. Methods

This manuscript is narrative review synthesizing evidence on post-COVID-19 condition (long COVID/PASC) in children and adolescents, with emphasis on epidemiology, clinical presentation, pathophysiological mechanisms, diagnostic considerations, and management. Although not designed as a systematic review, a structured search and predefined selection principles were applied to improve transparency and reproducibility. A structured literature search was performed across major biomedical and multidisciplinary databases, including PubMed/MEDLINE, Scopus, Web of Science, and Google Scholar. Searches were conducted for publications from January 1, 2020 onward, reflecting the emergence of SARS-CoV-2 and long COVID as a clinical entity. In addition, manual reference-list screening (snowballing) of key articles and reviews was undertaken to identify relevant studies not retrieved through database indexing, including early reports and consensus documents. Search terms were adapted to each database and combined using Boolean operators. Core concepts included long COVID terminology and pediatric population terms, for example: (‘long COVID’ OR ‘post-COVID condition’ OR ‘post-acute sequelae of SARS-CoV-2’ OR ‘PASC’ OR ‘post-COVID syndrome’) AND (‘child*’ OR ‘pediatric*’ OR ‘adolescent*’ OR ‘young people’ OR ‘youth’) AND, when relevant to specific sections, (‘symptom*’ OR ‘fatigue’ OR ‘cognitive’ OR ‘dyspnea’ OR ‘autonomic’ OR ‘POTS’ OR ‘endothelial’ OR ‘microclot*’ OR ‘inflammation’ OR ‘autoimmunity’ OR ‘rehabilitation’ OR ‘management’ OR ‘treatment’). To support a pediatric-focused synthesis, studies and documents were eligible if they met the following inclusion criteria: published 2020 onward; in English; addressed post-acute sequelae/long COVID/post-COVID condition following SARS-CoV-2 infection; included children/adolescents (≤18 years), or presented pediatric findings separately; study types: observational studies (cohort, case–control, cross-sectional), case series, clinical trials, systematic reviews/meta-analyses, consensus statements/guidelines, and major surveillance/statistical reports relevant to burden estimates. Exclusion criteria were applied as follows: studies limited to acute COVID-19 with no post-acute follow-up; studies where pediatric data were not separable from adult data; non-English publications; commentaries/opinion pieces without original data (unless used only for contextual framing); preclinical/animal/in-vitro studies without direct clinical pediatric relevance. Records retrieved from database searching and manual screening were evaluated for relevance by title/abstract screening, followed by full-text assessment for items judged potentially eligible. Because definitions of long COVID, follow-up timepoints, outcome measures, and comparator groups vary widely across pediatric studies, formal meta-analysis was not planned. Evidence was therefore synthesized descriptively and organized by clinical domains (e.g. symptom clusters, organ systems, and functional impact), with integration of consensus definitions and guidance documents when applicable. Across database searches and manual reference screening, a total of 345 sources were selected and cited in the manuscript reference list. These include primary pediatric studies, controlled and uncontrolled observational datasets, systematic reviews/meta-analyses, and authoritative guidance/consensus documents (e.g. WHO/NICE/CDC and national surveillance reports), supporting both pediatric-specific conclusions and mechanistic context.

## 3. Epidemiology of LC-19

The epidemiology of LC-19 in children and adolescents remains incompletely defined, with substantial heterogeneity in reported prevalence across studies. Variability reflects differences in study design, follow-up duration, symptom definitions, pandemic phase, circulating viral variants, vaccination status, and the inclusion or absence of control groups [25,26]. The actual rate of L-C19 in children varies by region and study [10–12,19, 27–38]. It has been estimated that 4% to 20% of children infected with SARS-CoV-2 will develop L-C19, with some studies showing even higher rates (66%), especially when including mild or asymptomatic cases [11,38]. Further follow-up of children after they had COVID-19 shows that some children still have symptoms for several weeks or months. For instance, a study conducted by the UK Office for National Statistics found that 2. Five% of children between the ages of 2 and 11 years and 5% of children between the ages of 12 and 16 years had L-C19 symptoms that lasted for at least eight weeks [29]. Several factors increase the risk of developing L-C19; however, the mechanism of how this occurs is not well understood [12,39]. Children who are 12 years old and above, especially those with comorbidities, are at a higher risk of developing L-C19 than younger children [40–43]. Some studies have revealed that girls may have a higher likelihood of developing L-C19 than boys, but the cause for this pattern is not well understood [9,38]. L-C19 can also develop even if the individual has mild or asymptomatic COVID-19; however, children who have more symptomatic or severe COVID-19 (including those who were hospitalized) are at a higher risk of developing long-term symptoms [44–46]. It has been suggested that children with other health conditions, including asthma, obesity, or immune system disorders, may be at higher risk of developing L-C19 [46–48]. Prolonged symptoms may be due to chronic respiratory or neurological chronic conditions [48,49]. Newer variants (such as Delta or Omicron) appear to cause different patterns of infection, and there may be differences in the risk of developing L-C19 with each variant. However, L-C19 has been observed in children affected by earlier forms of the virus, as well as newer versions [50].

## 4. Pathophysiology and immunological aspects

The mechanisms underlying L-C19 in children differ significantly from those in adults, primarily in terms of symptoms, pathophysiology, and prevalence. Pediatric L-C19 often presents with a broad range of symptoms affecting multiple organ systems, including respiratory, neuropsychiatric, and gastrointestinal issues, with notable symptoms such as fatigue, headache, and muscle weakness. In contrast, adults frequently experience severe respiratory and cardiovascular complications [51–53]. The prevalence of L-C19 in children is not precisely known, but it is estimated that a significant proportion of pediatric COVID-19 survivors experience persistent symptoms [54]. The management of L-C19 in children requires a multidisciplinary approach, focusing on individualized care to address diverse and overlapping symptoms [55]. Given the potential long-term health impacts, ongoing research is required to better understand the pathophysiological and immunological underpinnings of L-C19 in children, which will allow for more effective diagnostic and therapeutic strategies [54]. Several mechanisms have been proposed to be involved in L-C19 activity in both children and adults. First, autoimmunity and immune dysregulation are prominent hypotheses for both populations; however, children may exhibit unique autoimmune responses such as elevated antinuclear antibodies, which are less commonly reported in adults [51]. Additionally, children generally experience lower prevalence rates of L-C19 than adults, with studies indicating that teenagers are more affected than younger children [56]. Understanding these differences is crucial for developing age-appropriate diagnostic and management strategies [57]. Symptom patterns in children also differ by age, with school-age children being more likely to experience neurocognitive, pain, and gastrointestinal symptoms, whereas adolescents often report changes in smell or taste, pain, and fatigue [58]. Immune dysregulation is a prominent hypothesis, with studies indicating that children with L-C19 exhibit significant immunological differences compared with those who have fully recovered. These differences include skewed T and B cell subsets and an imbalance in regulatory T lymphocytes, suggesting a compromised ability to transition from innate to adaptive immune responses [59,60]. The persistence of viral reservoirs and the potential reactivation of other viruses, such as the Epstein-Barr Virus, may also contribute to prolonged symptoms [61]. Clinically, children with L-C19 experience a range of symptoms including respiratory issues, fatigue, cognitive disturbances, and musculoskeletal pain, which can significantly impact their daily activities and quality of life [51,57,62]. Endothelial dysfunction is a central feature of both acute COVID-19 and L-C19, characterized by damage to endothelial cells that line blood vessels, leading to increased permeability, inflammation, and a pro-thrombotic state [63–65]. In children, as in adults, the persistence of endothelial dysfunction after the acute phase of COVID-19 can contribute to long-term cardiovascular complications, including thrombotic events and microvascular damage [66,67]. The pathophysiological mechanisms involve a complex interplay of factors such as viral persistence, immune dysregulation, and the formation of fibrinoid microclots, which are resistant to fibrinolysis and can entrap inflammatory molecules, exacerbating endothelial damage [68,69]. The presence of elevated biomarkers such as von Willebrand factor and platelet factor 4 in L-C19 patients further underscores the role of endothelial activation and platelet hyperactivation in disease pathology [70]. These biomarkers indicate ongoing endothelial inflammation and a heightened state of coagulation, which can lead to microvascular thrombosis and contribute to the multisystemic symptoms observed in L-C19 [71]. Moreover, the persistence of endothelial dysfunction in L-C19 is not only a result of direct viral effects but also of secondary mechanisms such as autoimmunity and chronic inflammation, which can perpetuate endothelial damage and coagulation abnormalities [67]. This is particularly concerning in children, as their developing systems may be more vulnerable to the long-term effects of vascular and immune dysregulation.

## 5. Clinical manifestations

**Figure 2.**
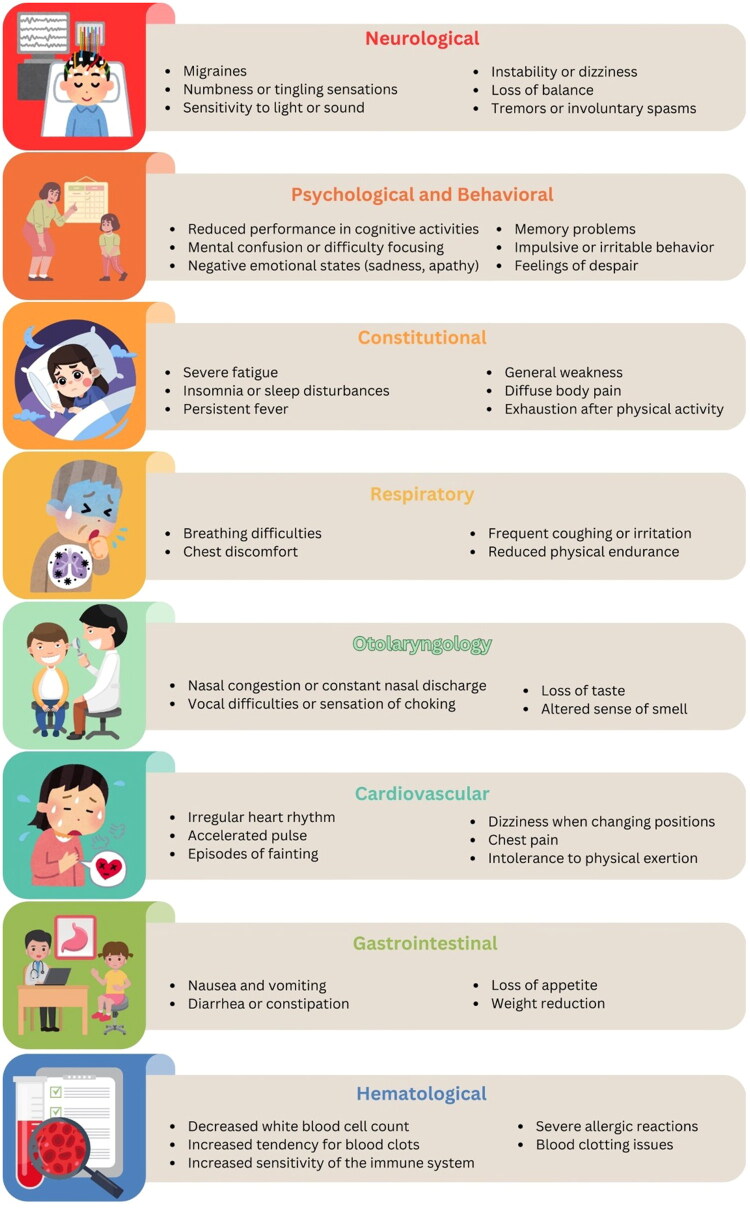
Main symptoms and signs that occur after SARS-COV-2 infection in children.

Most children with COVID-19 only get better and do not experience long-term effects, but any children who do get L-C19 can experience a variety of symptoms [72,73]. L-C19 in children includes a heterogeneous group of symptoms and conditions that appear after infection with SARS-CoV-2, which may reflect either the persistence of symptoms from the acute phase of the infection (cough, breathing difficulties, headaches, fatigue, loss of taste, and smell) or exacerbation of preexisting conditions after SARS CoV-2 infection (Figure 2). Thus, persistent cough in children with asthma and chronic lung disease, diabetic ketoacidosis in children with diabetes, and exacerbation of chronic neurophysical diseases have been reported [72,74,75].

PASC can also include some diseases that appear de novo after infection with SARS Cov-2, such as development of type 1 diabetes or autoimmune diseases [74,76]. One of the serious complications that can occur after SARS CoV-2 infection is the multisystem inflammatory syndrome in children (MIS-C), observed between 2 and 6 weeks after the initial infection [75]. Some of the most common symptoms reported in children with L-C19 include respiratory, otorhinolaryngologic, cardiac, constitutional, neurological, gastrointestinal, dermatological, musculoskeletal, and hematological manifestations.

### 5.1. Respiratory manifestations

**Figure 3.**
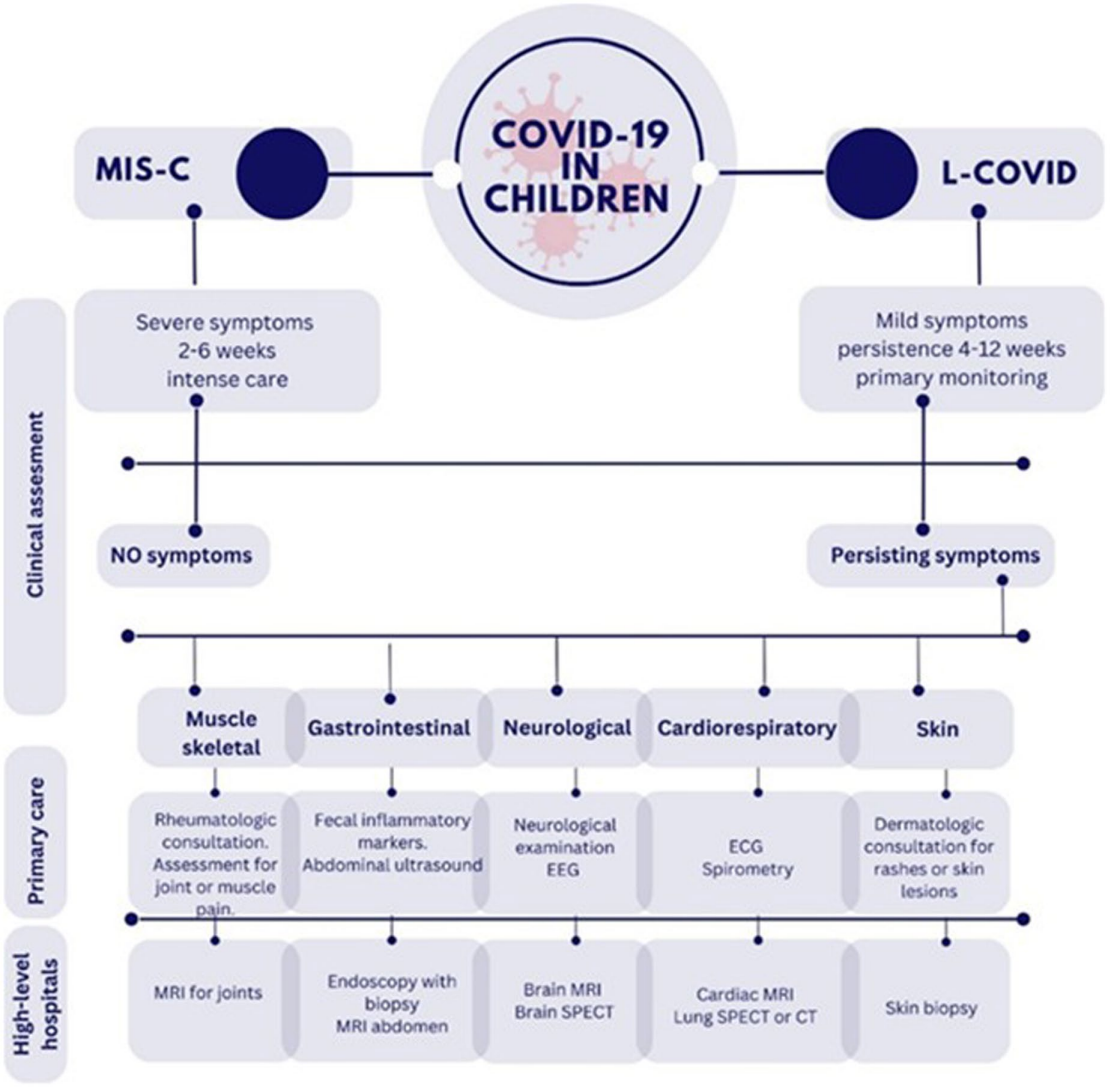
Management of main symptoms in L-C19 in children.

The common L-C19 symptoms in children include respiratory manifestations. Although respiratory issues are more frequently associated with the acute phase of COVID 19, some children have persistent respiratory symptoms long after the infection has cleared [77] (Figure 3). Although these ongoing symptoms can negatively affect a child’s ability to perform normal daily activities including physical activities, they often require ongoing medical follow-up. Some children with L-C19 said that they find themselves winded with little effort. It can occur as mild breathlessness or interfere more seriously with daily activities [78]. Physical exertion may worsen shortness of breath and reduce tolerance to exercise. After the acute phase of COVID-19, a dry cough (persistent or productive) can last for weeks or months [78,79].

Some children recover from their coughs within a few weeks, but others may experience chronic cough as one of the L-C19 symptoms. Children with L-C19 may feel chest tightness or sharp, stabbing chest pains [80]. Distressing for the child and parents, this symptom is not necessarily a cause of great concern if it is not accompanied by serious underlying problems such as myocarditis [81]. Inflammation within the lungs or chest wall may cause chest pain or trouble breathing [81]. Some children develop wheezing, which may indicate some degree of airway obstruction or inflammation in the lungs related to the effects of the virus on the respiratory system [73]. Other children may have symptoms of tachypnea, which can result from inflammation of the airways or long-term consequences of COVID-19 on lung function [80,82,83]. Children with L-C19 may find that they cannot keep up with physical activities that were no problem before. Physical exercise can lead to heightened respiratory distress, fatigue, and feeling out of breath, which make it difficult for them to play sports or physical play [83,84]. There is no clear picture of what causes respiratory symptoms in L-C19, although several factors may be involved. The inflammation caused by SARS-CoV-2 may continue in the respiratory tract even after the acute infection is over and can cause the person to develop chronic respiratory symptoms including coughing, shortness of breath, and wheezing [81]. In severe cases, COVID-19 can cause lung injuries. In children who didn’t have severe illness, even a mild inflammation or lung function changes could still be present to persist in symptoms [85–87]. After COVID-19 infection, some children develop oversensitive airways, causing asthma or bronchitis, and may result in wheezing, coughing, and trouble breathing [88,89]. The autonomic nervous system, which controls the body’s functions, such as heart rate and breathing, is also vulnerable to COVID-19. This system is dysfunctional and may lead to dysfunctional breathing patterns, such as shortness of breath [90].

The diagnosis of respiratory manifestations of L-C19 in children is based on clinical assessment, medical history, and diagnostic testing [88–91]. Diagnosis requires information on the child’s symptoms, when they start, and how they have continued once the child has recovered from COVID-19. Other causes of respiratory distress, such as asthma, allergies, and other viral infections, must be ruled out. Pulmonary function tests may be performed if respiratory symptoms are severe or persistent to test lung capacity, airflow, and the lung’s ability to function [83,88]. Chest X-ray or CT scans can help rule out structural lung damage and other COVID-19 related complications [92,93]. Blood tests such as C-reactive protein (CRP), lactate dehydrogenase (LDH), and erythrocyte sedimentation rate (ESR) can detect inflammation, and pulse oximetry can measure the amount of oxygen in the blood if respiratory symptoms affect this [94–98].

#### 5.1.1. Management of respiratory symptoms in L-C19

**Figure 4.**
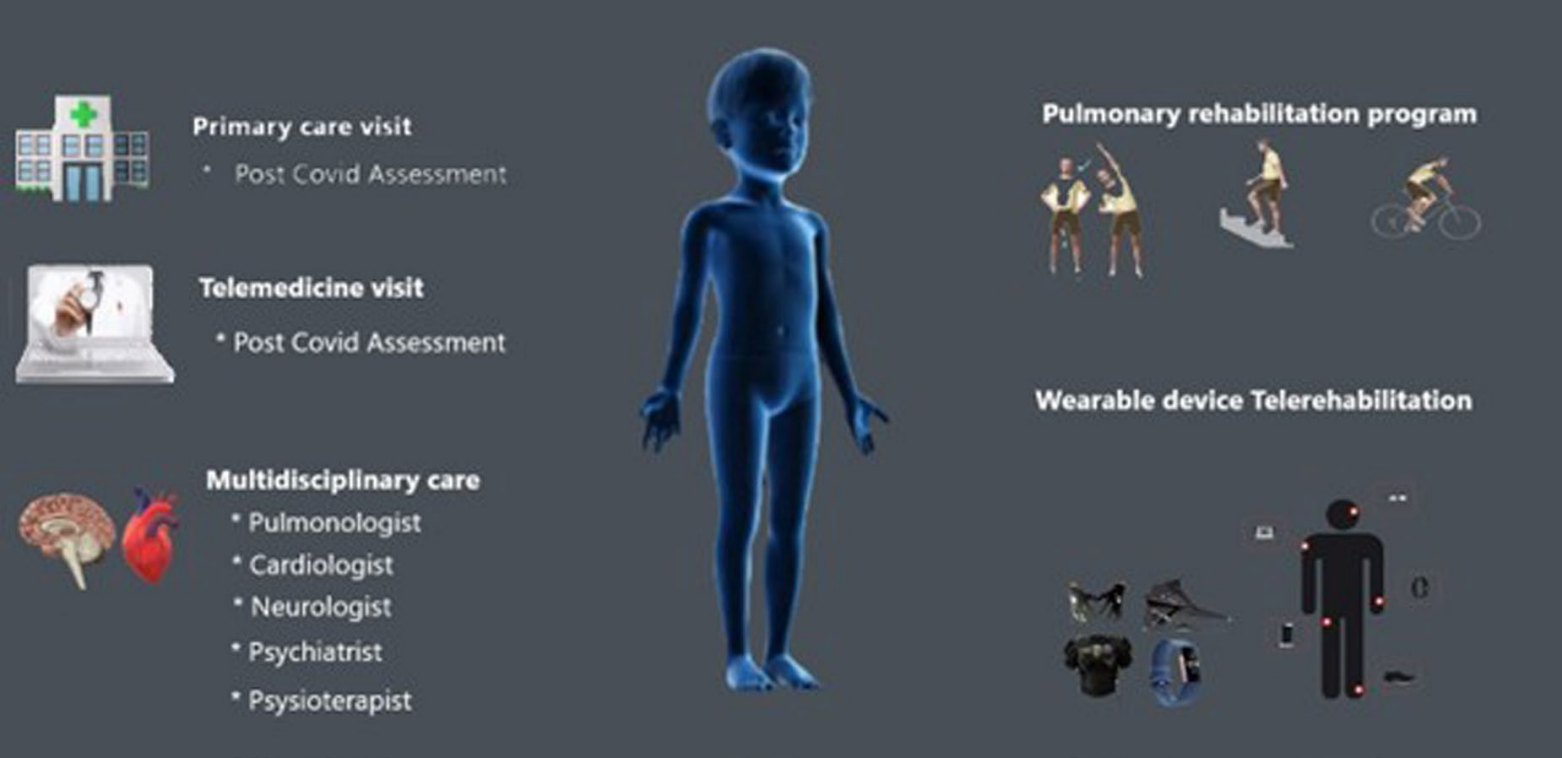
Management of Respiratory L-C19.

The treatment of respiratory manifestations of L-C19 in children includes symptom relief, improved quality of life, and prevention of further respiratory deterioration [99]. Bronchodilators (such as inhalers) are prescribed to help open the airways if there is wheezing or airway constriction, and to allow easy breathing [37]. However, in cases in which inflammation plays a major role in causing symptoms, corticosteroids or other anti-inflammatory treatments can be used to reduce airway inflammation [37,80,100,101]. Supervised physical activity and breathing exercises in pulmonary rehabilitation programs can help children regain strength and improve their exercise tolerance (Figure 4). Other treatments, depending on the child’s specific symptoms, include cough suppressants, pain relievers, or sleep aids that help the child manage the symptoms and get better sleep [102]. If children also have chronic respiratory symptoms, they can also contribute to anxiety or stress, which may require psychological support or counselling to help them deal with the emotional effects of L-C19 [100,102].

Respiratory symptoms are a significant aspect of L-C19 in children and can affect their quality of life. Persistent symptoms such as shortness of breath, cough, and chest pain require careful evaluation and management. Although many children recover over time, some may require long-term care to manage these ongoing respiratory issues (Figure 4).

### 5.2. Otorhinolaryngologic manifestations

L-C19 otorhinolaryngologic manifestations are defined as persistent or recurrent symptoms arising in the ear, nose, and throat (ENT) regions after infection with COVID-19 [9,38,39]. They are seen in both children and adult sufferers of L-C19 and can make a child’s life difficult and severely impair his or her ability to communicate, eat, sleep, and participate in daily tasks. Acute COVID-19 is characterized by the hallmark symptoms being loss of smell and taste. In addition, for some children, this may continue even after the acute phase of the illness [38,39,102]. Loss of smell and taste was one of the hallmark symptoms of acute COVID-19 infections. For some children, this may persist even after the acute phase of the illness has resolved. The sense of smell may return partially or be altered, leading to a distorted sense of smell or taste. Parosmia and dysgeusia can make food and other experiences unpleasant. In most children, these symptoms improve over time, although in some children they can last for several months [103]. The otorhinolaryngologic manifestations of L-C19 in children can consist of chronic nasal congestion or blockage. This manifestation can appear even after the acute viral infection has subsided in the child, and can cause problems with breathing, sleep, and overall comfort. Sometimes post nasal drip causes throat irritation or coughing [104]. Persistent or recurrent sore throat is a problem in some children who have L-C19. It can result from throat irritation, post-nasal drip, or even lingering viral infection. It may be a mild and sometimes intermittent sore throat, but it can be bothersome and interfere with the child’s ability to eat, speak, or sleep. Some children may have ear fullness or even mild ear pain, perhaps due to fluid building or Eustachian tube dysfunction [105]. Some children with L-C19 have heard of ringing or buzzing in their ears, a condition known as tinnitus. This can be distressing and may worsen with exposure to loud noise or stress. The mechanism of tinnitus in L-C19 is not yet known, but it could be due to viral effects on the auditory pathways or inflammation of the structures that interact with the ear [106–108]. Difficulty or discomfort in swallowing may be an otorhinolaryngologic symptom of L-C19 in children. This may be due to the virus irritating or inflaming the throat, post nasal drip, or acid reflux. Dysphagia can produce the sensation of food being stuck in the throat, causing difficulty with eating, and can eventually lead to difficulties with nutrition due to poor appetite and avoidance of eating [36,38]. This dysfunction in the sense of taste and smell is due to the direct effect of SARS CoV-2 on nerves [43]. These symptoms persisted beyond the course of acute infection, suggesting that they may be due to ongoing viral effects or host immune responses. The virus can cause inflammation in the upper respiratory tract, which lasts long after acute infection is over [43]. These symptoms can persist for several weeks, and may include continuous nasal congestion, sore throat, and ear fullness. An exaggerated or dysregulated immune response may last long and affect the mucous membranes of the nose, throat, and ears, causing chronic symptoms such as nasal congestion or sore throat [44]. The sensation of ear fullness, pain, and hearing problems may also be due to inflammation, fluid buildup in the middle ear, or Eustachian tube dysfunction [107]. The persistence of changes in smell and taste may be attributable to changes in brain processing of sensations. Anosmia or dysgeusia may persist because L-C19 alters the sensory signal processing of the brain [43,44].

Children with persistent otorhinolaryngologic symptoms may be referred to an otorhinolaryngologist (an ENT specialist). An examination of the nose, throat, and ears will be performed to identify signs of inflammation, fluid buildup, or other abnormalities. In certain instances, an endoscopic examination of the upper airways or sinus cavities will be performed to determine whether there are structural problems combined with infection or inflammation, which may be the cause of the symptoms. Specialized tests may be performed to assess the function of the olfactory and gustatory systems to evaluate the existence of abnormalities in smell and taste. Hearing function can be assessed, and hearing loss or other L-C19 problems with hearing can be ruled out using audiometric testing [105–107].

#### 5.2.1. Management of otorhinolaryngologic symptoms in L-C19

Management of otorhinolaryngologic manifestations in children with L-C19 involves symptom management and improves their quality of life. Smell training is recommended for children with chronic, persistent anosmia or exposure of the child to various scents for patients with dysgeusia, in order to retrain the olfactory system [92]. If nasal congestion or blockage is troublesome, it may be useful to prescribe decongestants or nasal corticosteroids that will reduce inflammation and open up the nasal passages [44]. Saline solutions often rinse nasal passages to help clear congestion and improve the flow of air. Sore throat symptoms may be soothed with over-the-counter remedies, such as throat lozenges and warm saltwater gargles, and in certain instances, they may need to take an anti-inflammatory effect to reduce irritation. If there is ear fullness or hearing trouble, ear exercises or decongestants may relieve symptoms. Hearing aids can be provided in more severe cases. Speech therapy is performed for children with trouble swallowing (dysphagia) or changes in vocalization [106,107]. In children, L-C19 manifestations, such as anosmia, nasal congestion, sore throat, and ear-related symptoms, become a major part of otolaryngologic symptoms. These symptoms make it difficult for them to significantly alter their daily activities, eating habits, and social interactions. Long-term symptom management and recovery in children is achieved using a multidisciplinary approach to diagnosis and management, including ENT evaluation, symptom relief, and rehabilitation strategies.

### 5.3. Cardiac manifestations

Pediatric cardiac complications of L-C19 are not as frequent as other signs and symptoms, including fatigue and respiratory problems [31]. However, some children do develop persistent or new-onset cardiac issues after their initial COVID-19 illness. These manifestations can directly impact the heart and, therefore, may have different complications that require further observation and intervention [99–118]. The most common cardiac symptom reported in children with L-C19 is chest pain [38]. In fact, a large meta-analysis found chest pain to be the most common cardiovascular symptom among long-COVID patients overall [119]. Children can experience this pain as sharp and stabbing or aching and dull and can feel it occasionally or all the time. The distress and discomfort of pain, however, may not always mean serious heart disease, but it can be distressing and uncomfortable. In most cases, it is believed to be associated with inflammation or musculoskeletal problems rather than actual heart damage [108,109]. Abnormal awareness of the heartbeat, such as feeling heart racing or pounding, is called palpitations [27]. Children with L-C19 may experience episodes of an unusually fast heart rate or even transient arrhythmias [38,111]. Some develop a persistent tachycardia, meaning their resting heart rate remains elevated well after recovering from the acute infection [109,110,117]. This persistent high heart rate is thought to be related to COVID-19–induced autonomic dysfunction, where the nervous system’s regulation of heart rhythm is disrupted [120]. Palpitations and tachycardia in these children often worsen with physical activity or emotional stress, and the episodes can be accompanied by dizziness, lightheadedness, or occasional fainting [118]. The intensity of these symptoms can understandably provoke anxiety in affected children and their families [112]. Less common than tachycardia, L-C19 sometimes causes bradycardia when the heart beats slower than it should [117]. Although dyspnea is usually a respiratory symptom, when a child also complains of palpitations or other cardiac symptoms, it can also be suggestive of cardiac involvement [121]. A reduced heart’s ability to pump effectively can cause shortness of breath due to pulmonary congestion or lack of oxygen in tissues. Many children have L-C19, and report an inability to participate in physical activities at the normal level. This exercise intolerance stems not only from post-viral fatigue and muscle weakness, but also from the cardiac symptoms described above [106,107] Physical exertion may increase heart rate, dizziness, and shortness of breath in children, making it difficult for them to participate in sports, physical education, or other forms of exercise [117,118,122].

Myocarditis is a notable, though uncommon, complication observed in pediatric and young adult COVID-19 patients. Myocarditis can occur during COVID-19 infection, but it can also occur in the post-COVID-19 period as one of the L-C19 symptoms [110,115]. Children with myocarditis may present with symptoms like chest pain, difficulty in breathing, palpitations, and fatigue. If left untreated, myocarditis can lead to serious outcomes such as arrhythmias, heart failure, or even death in severe cases. Like myocarditis, pericarditis can occur either during the acute illness or later in recovery [110,115,122]. It typically causes chest pain accompanied by fatigue and shortness of breath. Both myocarditis and pericarditis require prompt medical attention, as they can cause lasting damage to cardiac tissues if severe, although most pediatric cases are mild and tend to improve with appropriate treatment. As a result of autonomic dysregulation caused by COVID-19, the autonomic nervous system (ANS) can be affected. Involuntary functions, such as heart rate, blood pressure, and digestion, are controlled by the ANS. Abnormal heart rhythms, a hard time regulating blood pressure and a ‘fluctuating’, heart rate can result from dysregulation of this system [123]. As part of the ANS disruption in LC-19, some children develop a condition known as postural orthostatic tachycardia syndrome (POTS) [124]. POTS is characterized by an excessive increase in heart rate when moving from sitting or lying down to standing up, often accompanied by symptoms of orthostatic intolerance. A child with POTS may experience marked dizziness, lightheadedness, or even fainting upon standing, along with chronic fatigue. They can also develop various other symptoms such as chest pressure, ongoing palpitations, cutaneous vasodilation, excessive sweating, and tremors in the limbs [124]. While not life-threatening, POTS can significantly interfere with daily activities. Notably, pediatric POTS symptoms such as dizziness, lightheadedness, and exercise intolerance overlap with many long COVID complaints. Fortunately, severe long-term cardiac outcomes remain rare in pediatric L-C19. In very uncommon instances, however, persistent cardiac inflammation (for example, from significant myocarditis or pericarditis) can lead to heart muscle damage and subsequent heart failure [122]. Signs of heart failure include extreme fatigue, fluid retention (edema), and troubled breathing, especially with exertion or when lying down [122].

The pathophysiology behind these long-term cardiac manifestations is still being investigated. SARS-CoV-2 can directly infect cardiac tissue, causing inflammation of the myocardium or pericardium that may persist beyond the acute illness. In addition, the body’s immune response after the virus is cleared can sometimes remain overactive, leading to chronic inflammation that affects the heart [124]. This post-viral inflammatory state is one explanation for ongoing symptoms such as tachycardia, chest pain, and rhythm disturbances seen in L-C19 patients [122–124]. COVID-19 has also been linked to autonomic nervous system dysfunction, as noted above, which provides another mechanism for symptoms like inappropriate sinus tachycardia, intermittent bradycardia, and the development of POTS [90,123]. Furthermore, SARS-CoV-2 infection is associated with endothelial dysfunction which can impair normal blood flow and blood pressure regulation. All these factors together may contribute to the cardiovascular problems observed in children recovering from COVID-19.

The diagnosis of cardiac symptoms in children with L-C19 can be made through clinical assessment, obtaining medical history, and performing diagnostic tests, such as the time when the symptoms appeared and whether there was any link with the original child’s COVID-19 infections [83,121–127]. An electrocardiogram (ECG) can be used to determine heart rhythms and to look for arrhythmias, tachycardia, bradycardia, or electrical disturbances in the heart [123]. ECG is an important diagnostic test that allows the evaluation of heart function, as well as the detection of any structural abnormalities, myocarditis, or pericarditis. In more severe cases, cardiac Magnetic Resonance Imaging (MRI) may be used to detect myocarditis, pericarditis, or other forms of heart damage that an echocardiogram may not visualize [124,125]. Cardiopulmonary exercise testing (CPET) and/or ventilation–perfusion single-photon emission computed tomography (V/Q SPECT) can identify pulmonary circulation dysfunction. Blood tests to verify markers of inflammation, such as C-reactive protein, troponins, N-terminal pro B-type natriuretic peptide (NT-BNP), or BNP, may be performed to determine if the heart is involved [117,122]. Complete blood count, liver, kidney, and thyroid function tests, and glycosylated hemoglobin could be performed when an alternative diagnosis was suspected. POTS can be diagnosed using the active standing test in patients presenting with orthostatic intolerance (OI) and a normal heart rate (HR) in the supine position. HR can increase by at least 40 beats per minute (bpm) in the first 10 min of this test, with a maximum HR of 130 bpm for 6- to 12-year-olds and over 125 bpm for 13- to 18-year-olds [83,126]. Another diagnostic test is the ‘passive head-up tilt test (HUTT) ‘, which can show a drop in systolic blood pressure of > 20 mmHg or a drop in diastolic blood pressure of > 10 mmHg. Quantitative sudomotor axonal reflex testing (QSART) and increased serum TNF-α levels are useful for the diagnosis of POTS, suggesting an immune-mediated mechanism in the occurrence of this post-COVID syndrome in children [127].

#### 5.3.1. Management of cardiac symptoms in L-C19

**Figure 5.**
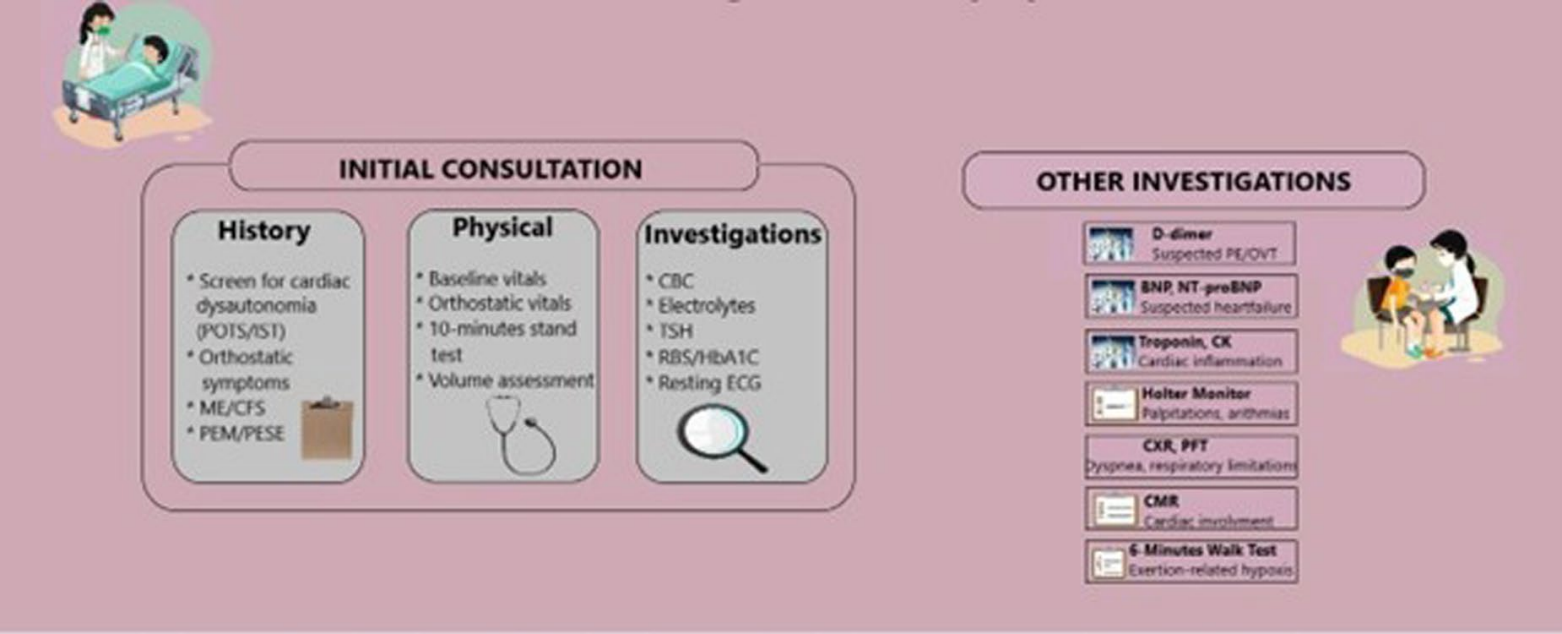
Management of cardiac L-C19 in children.

Treatment of the cardiac symptoms of long COVID in children focuses mainly on relief of symptoms, monitoring for complications, and promoting recovery (Figure 5).

Medications may help control symptoms, such as palpitations, tachycardia, or chest pain [127,128]. Elevated heart rates and arrhythmias may be controlled using beta-blockers or other heart rhythm medications [128]. Slow steady progression back to physical activity with close observation of the heart rate and symptoms is often part of the recovery process. The non-pharmacological treatment of POTS consists of establishing a diet with high sodium content (10–12 g/day) and increased fluid intake (2–3 L/day), exercises (isometric, aerobic, and resistance exercises), compression garments, and avoiding prolonged orthostatism [129–131]. Pharmacological treatment consists of the administration of small doses of beta-blockers (propranolol and metoprolol), fludrocortisone, α-receptor agonists (midodrine), and a selective sinus node inhibitor (ivabradine) in combination with a high-sodium and omega-3 fatty acid diet for orthostatic symptoms [129,132]. Cardiac rehabilitation can be useful in children to enhance their ability to exercise and improve their cardiovascular health [131]. Patients with cardiac symptoms should be followed-up by pediatric cardiologists frequently if they have myocarditis, pericarditis, or autonomic dysfunction. Follow-ups may involve repeated ECGs, echocardiograms, and other tests as well [133]. Some cardiac symptoms can cause anxiety or stress, particularly in children. Therefore, other psychosocial interventions such as counselling or therapy may be required to assist children in dealing with the psychological impact of L-C19 [134].

### 5.4. Constitutional symptoms

L-C19 constitutional symptoms in children are systemic symptoms throughout the body that are often non-specific and can be observed in multiple medical conditions. These symptoms are more common than persistent or recurrent and result in difficulties with normal activities, social interactions, and quality of life. L-C19 can sometimes include constitutional symptoms as its first symptoms, which can be difficult to manage because they can fall anywhere [31,35]. The most commonly reported symptom of L-C19 in children is fatigue [19,36]. It is more than normal tiredness, and can keep a child from doing day-to-day things, going to school, or playing physical activities [135,136]. Fatigue in L-C19 patients is often severe and can last for weeks or months after the original infection ends [104]. This can be variable, with variations in severity, and may worsen as a result of exertion, a process called ‘post exertional malaise’ [137,138]. Fever is a hallmark of the acute phase of COVID-19, but children with L-C19 may continue to develop intermittent or low-grade fever after recovery from the initial infection [136]. It is a persistent fever that may start and stop without any identifiable trigger [36,37]. Other symptoms, such as chills, body aches, and headaches may be present along with fever [9,36,38]. Frequent constitutional symptoms in children with L-C19 are myalgia and arthralgia [37,139]. The intensity of these pains can vary, and they usually affect the limbs, back or neck [139]. Children may have stiffness, soreness, or trouble moving a joint, which may cause discomfort and limit physical activity [38,39,139,140]. L-C19 headache is common in children. Headaches can be mild to severe, and may also be sensitive to light or sound. The cause of these headaches is still unclear, but they may be due to neurological changes, inflammation, or the body’s immune reaction to the virus [141]. Sleep problems such as insomnia, trouble falling asleep, and waking often during the night are common in children with L-C19. This may be due to anxiety, fatigue, or other physical factors, such as muscle or joint pain, which makes it difficult to do nothing [5,134].

Some children say that they sweat excessively, even during the night, in a room that feels comfortable. It sometimes has to do with the body’s effort to regulate its temperature, and at other times, it means the temperature is a way the body is telling you there is an ongoing inflammation or infection [5]. Malaise is defined as a vague sense of discomfort, wellness, or illness that often includes fatigue. Without these physical symptoms, children may still feel unwell or like they do not have as much energy [5,141]. Some pediatric patients with L-C19 develop lymphadenopathy. Persistent immune activation of the virus can cause lymph nodes to become enlarged, particularly in named areas such as the neck, armpit, or groin [5,142]. Even when not considered a constitutional symptom, brain fog (cognitive symptoms of difficulty concentrating, memory problems, and feeling mentally tired) is often reported in children with L-C19. This can come in the form of difficulty focusing on tasks, feeling mentally ‘foggy’, or being unable to do schoolwork, or otherwise complete a cognitive task [143]. The exact cause of the constitutional symptoms of L-C19 in children remains unknown, but there may be several explanations. COVID-19 infection may lead to prolonged inflammation in different tissues, including the muscles, joints, brain, and nervous system [84]. However, this inflammation can also cause a person to feel fatigued, experience headaches, muscle and joint pain, or malaise. Immune dysregulation is believed to be involved in L-C19, in which the body’s immune system continues to be triggered after the virus has been cleared. Consequently, inflammation and symptoms such as fever, lymphadenopathy, and fatigue can persist [85]. COVID-19 can disrupt the ANS, an involuntary system for governing factors, such as heart rate, digestion, and body temperature. Dysfunction in the ANS may lead to symptoms including fatigue, inability to regulate temperature, and difficulty sleeping [90]. With L-C19, enduring effects on the nervous system may result in enduring symptoms, such as cognitive dysfunction, headaches, and malaise. While some symptoms dissipate once a virus leaves the body, the brain’s response can cause lingering symptoms [144,145]. Post-exertional malaise (PEM) can be defined as worsening of previous symptoms after engaging in physical or mental activity. In children with L-C19, a notable worsening of symptoms is seen after physical or cognitive activity, which leads to prolonged fatigue, myalgia, and other constitutional symptoms [146,147].

Constitutional symptoms in children with L-C19 can be diagnosed by a review of the child’s signs and symptoms, the timeline of the symptoms, and whether the child has had a recent COVID-19 infection or diagnosis [148]. It is equally important to determine the duration of the symptoms and exclude other possible causes of the child’s discomfort. Fatigue symptoms in patients with L-C19 can be assessed through self-reporting or measured using fatigue assessment tools such as the Fatigue Severity Scale (FSS) and the Chalder Fatigue Scale (CFs) [10,149,150]. Blood tests may include inflammatory markers, such as C-reactive protein, erythrocyte ESR, or other abnormalities that could account for constitutional symptoms [151,152]. Because L-C19 can resemble or overlap with other medical conditions, other possible causes for the child’s symptoms, such as autoimmune disease, another viral infection, or chronic fatigue syndrome, also need to be ruled out. They are recommended for the differential diagnosis of other diseases, muscle enzyme testing (creatine kinase, lactate dehydrogenase, and myoglobin), ionogram, complete blood count, evaluation of liver and kidney function, thyroid function tests, rheumatoid factor, and antinuclear antibodies [151–153]. In some cases, it may be necessary to perform electromyography (EMG), computed tomography, or MRI to assess the presence of anatomical abnormalities in the musculoskeletal system, functional assessment through the sit-to-stand test, or six-minute walk test (6 MWT) [154].

#### 5.4.1. Management of constitutional symptoms in L-C19

Fatigue is a common issue, and proper rest and healthy activity levels are all treatment strategies. Some children may have to pace themselves and avoid over-exertion to avoid post-exertional malaise [155]. Nonsteroidal anti-inflammatory drugs such as acetaminophen or ibuprofen can be used to relieve muscle or joint pain or headaches. Some ways to minimize sleep problems include following a routine schedule and ensuring that the sleeping environment is comfortable [156]. Techniques of relaxation or cognitive-behavioral therapy for insomnia (CBT­I) may also be helpful [157]. Children with severe fatigue or cognitive impairments may need to increase their physical activity and cognitive functioning little by little [158]. Rehabilitation processes can assist children in regaining their strength, endurance, and mental alertness. Treatments to rehabilitate muscle tone, gait, balance, water therapy, yoga exercises, and physical therapy can be important in the treatment of chronic fatigue from long-term COVID [148]. Psychological interventions, such as example counselling or psychotherapy, may be useful for children to assist them in dealing with the psychological sequelae of L-C19, including anxiety and depression, which may be compounded by constitutional symptoms.

### 5.5. Neurologic manifestations

Neurological manifestations in children with L-C19 indicate that one or more nervous system symptoms last for more than four weeks after the acute phase of COVID-19 [159]. The effects of these neurological problems on a child’s quality of life can be severe and may include cognitive, sensory, motor, and autonomic symptoms [160]. Not all children with L-C19 have neurological manifestations; however, when they do, it can lead to problems in learning, daily activities, and mental health. One of the most often reported neurological symptoms of long COVID in children is brain fog [11,136]. Children may have difficulty concentrating or recalling things, and generally suffer from mental confusion or ‘cloudiness’ [161,162]. Problems can manifest in a child’s ability to pay attention or focus, process information, and so it can affect a child’s academics and ability to function daily [160,161].

Children with L-C19 often experience headaches that differ in how much pain they cause, how often they happen, and how long they last [35,163–165]. These headaches can be tension type or similar to migraine and are often accompanied by additional symptoms, such as nausea, photophobia, or phonophobia [162,163]. They can be debilitating to children, making them unable to function in school or physical activities and causing headaches (sometimes daily or intermittently). A major complaint among children with L-C19 is dizziness or a sense of lightheadedness [141,166–172]. Vertigo, a spinning sensation, can also occur in some children and may be the result of changes in position or head movement [33,43,171]. Often associated with such problems in the balance system, these symptoms may be due to autonomic dysfunction or changes in the inner ear that result from the virus [173]. Neuropathy (a feeling of tingling, numbness, or pain in the extremities, such as the hands, feet, or legs) is possible in children with L-C19 [166]. These sensations can be intermittent, persistent or unpleasant. Inflammation or damage and subsequent peripheral neuropathy can occur due to immune system dysregulation or viral persistence in nerve tissues [42,104]. Sleep disturbances can also be caused by neurological impacts, such as falling asleep, frequent wakening, or poor sleep quality [31,35,43,104,166]. This often results in symptoms such as brain fog, headache, and anxiety [145]. Other L-C19 symptoms can be worsened by sleep problems, and recovery can be made more difficult by sleep disturbances [141]. Loss of smell and taste are typically acute symptoms of COVID-19, but some children with L-C19 experience persistent or delayed recovery of these senses; loss of smell and taste can affect a child’s appetite and quality of life [167,168]. These senses may return slowly in some cases, but recovery is slower in others [104,109]. Although quite rare, a few children with L-C19 have been reported to experience seizures [174]. However, these can appear as convulsions or other strange brain activities [160,166]. L-C19 can also affect a child’s mood and behavior [171]. Anxiety, depression, or irritability in children may be compounded by the stress of living with ongoing physical health problems [11]. The neurological effects of L-C19 can also lead to mood swings, irritability, or emotional dysregulation [33]. Some children with L-C19 may have difficulty in coordination or balance or may develop motor dysfunction. This can cause unsteadiness, problems with walking, and fine motor tasks (e.g. writing or buttoning a shirt) [37,38]. Inflammation or irritation of the brain or nervous system can cause motor symptoms, muscle weakness, and fatigue [37]. Persistent ringing or buzzing sounds in the ears (tinnitus) have been reported in some children with L-C19. Tinnitus is distressing and can occur in association with other symptoms of neurological involvement such as dizziness or headaches [44].

The mechanism by which COVID-19 affects neurological symptoms in children remains under investigation [175,176]. It seems that L-C19 could also induce ongoing inflammation in the brain and nervous system [177,178]. It can also disrupt normal brain function and cause mood, headache, and cognitive changes [179]. Some children can have an immune response after infection, which continues to attack body tissues such as the nervous system [145,177]. Immune dysregulation may manifest symptomatically as brain fog, neuropathy, or seizures [180]. The virus is less common and may directly invade neural tissues and cause damage to brain or nerve cells. The virus can spread to the central nervous system (CNS), which includes the brain, and possibly leads to long-term neurological symptoms such as memory issues, motor issues, or mood changes [177,178]. However, in severe cases of COVID-19, acute infection can cause low oxygen levels. Even after recovery, extended periods of low oxygen can cause permanent damage to the brain and nervous system, which is linked to L-C19’s cognitive and motor issues [181]. Some researchers have proposed that SARS-CoV-2 infection causes the blood to clot differently. This could affect the brain or peripheral nerve microclots or abnormal blood flow and cause neurological symptoms such as headaches, dizziness, or stroke-like symptoms [182].

Neurological symptoms in children with L-C19 are diagnosed using a combination of clinical assessment, symptom reporting, and diagnostic testing. Attention is paid to when neurological symptoms first appear, and if they are persistent or intermittent. A full neurological examination is performed to verify reflexes, coordination, balance, strength, sensation, and mental function [183]. However, brain imaging techniques, such as MRI or CT, may rule out structural abnormalities, brain inflammation, stroke, or other neurological conditions [184,185]. An electroencephalogram (EEG) may be used to monitor electrical activity in the brain and detect abnormal electrical activity patterns as an indication that seizures occur or to rule out epilepsy or other seizures [166,174]. In a few cases, if there are signs of infection or significant inflammation, cerebrospinal fluids (CSF) will be assessed for viral or inflammatory markers in the central nervous system [186]. Assessment of autonomic nervous system function, when suggested by symptoms of autonomic dysfunction (e.g. dizziness, fainting, and POTS), can be performed using tests such as heart rate variability, tilt table testing, and blood pressure monitoring [187].

#### 5.5.1. Management of neurologic symptoms in L-C19

**Figure 6.**
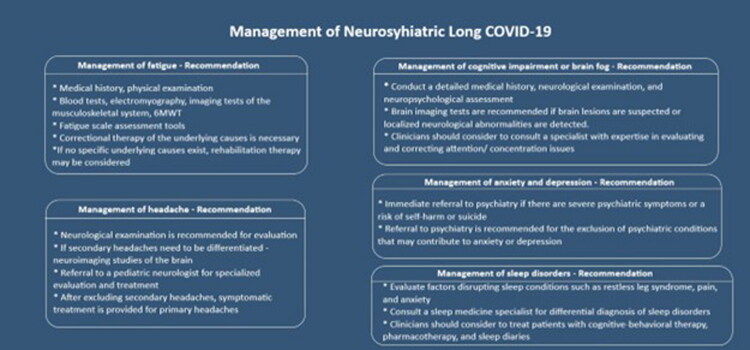
Recommendations regarding the management of the main neurologic symptoms.

Managing neurological symptoms is usually a multidisciplinary effort [155,188]. Symptom-oriented drugs, such as analgesics, anti-depressants, antianxiety medications, or painkillers, may be administered [148] (Figure 6).

Cognitive behavioral therapy (CBT) is effective for the treatment of ‘‘brain fog’’ and other cognitive dysfunctions. Cognitive rehabilitation may also be useful for children with attention and memory problems [160]. For children with motor concerns, difficulties with coordination, or neuropathy, physical therapy (PT) and occupational therapy (OT) can be beneficial for enhancing motor coordination, balance, and coordination [102,148]. Those who have experienced dizziness or vertigo might benefit from vestibular rehabilitation. Sleep disturbances can be treated with good sleep hygiene [189]. The best ways to improve rest and minimize fatigue include encouraging a consistent sleep schedule and creating a comfortable sleep. Such children may be helped by cognitive strategies or adjustments at school if they experience cognitive challenges such as memory or concentration problems [33]. Children who deal with anxiety, depression, or stress associated with L-C19 need ongoing emotional and psychological support [178]. Help is available from therapy, counselling, and peer support groups. Children with L-C19 should be encouraged to return to school, extracurricular activities, and sports, depending on their symptoms and how much energy they have. The concepts of pacing and energy conservation are important for avoiding the worsening of symptoms [156,189–191].

### 5.6. Mental health conditions

The prevalence of mental health disorders in children with L-C19 infection is a major issue. Some psychological problems include changes in the child’s emotional state and social relations as a result of prolonged physical and neurological consequences of L-C19 [192–194]. These mental health problems may be worsened by the chronic nature of the disease, its effects on schooling, and even the uncertainty of the cure. Anxiety, depression and other mood disorders are the most frequently observed mental health issues in children with L-C19 [171,195,196]. Generalized Anxiety Disorder (GAD) can be a manifestation of mental health disorders. Children with L-C19, who can still display symptoms months later, typically have long-term concerns about their health, recovery, and worsening [11]. Relating to their school performance, social interactions, and ability to relax or engage in leisure activities, children may excessively fear or be overly anxious about their physical health [33]. Even if they do not have serious underlying health problems, they may obsess symptoms or worry about new or recurrent health problems. Long-term illness and isolation through recovery can increase social anxiety. About one in three children say that when they return to school or social events, they will feel uncomfortable and fear judgment or health complications [197].

The risk of L-C19 in children is greater for depression, including lasting feelings of sadness or hopelessness, or losing interest in things they once enjoyed [198]. Symptoms include irritability, changes in appetite, difficulty sleeping, fatigue, and difficulty concentrating [199,200]. Children become depressed in ways different from adults, and it is difficult to diagnose. The way that L-C19 disrupts day-to-day life, schooling, and social activities can lead to feelings of hopelessness, frustration, and despair [192,201]. Children who were sick enough to require hospitalization for COVID-19 had a significantly increased risk of developing post-traumatic stress disorder (PTSD) and other poor mental health outcomes. One of the traumatic experiences is being seriously ill, which could result in severe medical procedures or lengthy hospital stays, bringing flashbacks, nightmares, and anxiety [18,202,203]. Hospital-related PTSD can manifest as avoidance of medical care, heightened startle responses, and ongoing fear that something terrible will happen again. Studies also suggest that parents of children hospitalized with COVID-19 have shown elevated PTSD symptoms, indicating how traumatic the pediatric COVID experience can be for the whole family [204]. Identifying PTSD in children is crucial, as it can severely impair their concentration, sleep, and mood. Trauma-focused therapies (like child-centered PTSD counseling) may be needed alongside medical follow-up. Difficulties with concentration and focus with symptoms called ‘brain fog’ have been reported in many children with L-C19 [136]. One may be especially obvious in schools, where children could have problems either finishing work or focusing in class. These symptoms are not, by any means, indicative of ADHD; instead, there are many cognitive dysfunctions and attention issues in long COVID. Children may present behaviors such as ADHD, including forgetfulness, distractibility, and trouble completing tasks [18]. However, in the context of long COVID, such symptoms are typically part of a broader post-viral cognitive dysfunction, not a new onset of ADHD. Unlike true ADHD (a developmental condition), long COVID ‘brain fog’ often comes with other fatigue and physical issues, and it gradually improves as the child recovers [205]. One symptom shared by children with L-C19 is mood swings that often occur because of frustration and emotional toll of living with symptoms that will not go away [206]. These can manifest as irritability, frustration, and even anger, far out of proportion to anything happening there. L-C19 also causes physical and cognitive stress in children, which can make it difficult to regulate their emotions. This occurs in relationships with family, peers or teachers [201]. L-C19 can contribute to the onset or intensification of obsessive-compulsive disorder (OCD) in children [33]. They may start having thoughts that are difficult to erase about their health, cleanliness, or even the chance of getting sick again, which may make them perform compulsive activities such as hand washing, checking, or avoiding certain places. These OCD symptoms are distressing for the child and family, and can lead to further anxiety, social withdrawal, or family conflict [207]. For example, a child might isolate themselves in their room to avoid germs or insist that parents follow elaborate cleaning routines. In the context of LC-19, OCD-like symptoms might be part of a broader post-infectious syndrome; some experts have speculated about links between COVID-19 and pediatric acute-onset neuropsychiatric syndrome (PANS), in which infections trigger sudden OCD symptoms [208]. School-related anxiety is seen in many children with L-C19 and manifests itself in the form of avoidance behaviors, panic attacks, and reluctance to return to school. Generally speaking, anxiety is the fear of being overwhelmed by the school environment, academic performance, or physical symptoms while at school. Anxiety can be exacerbated by pressure to keep up with homework and brain-related issues such as mental fog [197]. Social isolation for many children with L-C19 has increased because of prolonged illness and school closures during the pandemic. Because of ongoing symptoms or changes in emotions such as depression or anxiety, children may steer clear social activities or avoid friends and family. Withdrawal itself can contribute to feelings of loneliness, but it often worsens other mental health problems, such as depression or anxiety [159].

There are several reasons why children with L-C19 develop mental health conditions. Ongoing physical symptoms such as fatigue, headache, and muscle pain can cause helplessness, frustration and anxiety [192]. These frustrating symptoms can interfere with a child’s day-to-day routine and make the child feel out of sorts and unsure about the recovery process. Many children with L-C19 have been socially isolated for a long time due to persistent illness, missed school, and low social activity. Social withdrawal can cause feelings of loneliness, sadness, and anxiety, which can lead to mental health conditions [192,202]. L-C19 can severely interrupt schooling and adversely affect children’s academic performance, resulting in them feeling inadequate, stressed, and anxious. Children with this often have brain fog, concentration problems, and fatigue, which can make schoolwork difficult and frustrating, if not impossible [18]. L-C19 recovery is uncertain and, as a result, health anxiety may increase. Children may be scared that their symptoms will never improve, or they will get some new symptoms. They may also be anxious about being reinfected or suffering long-term effects of COVID 19. These first COVID cases can lead to PTSD in children who are sick or ill enough to be hospitalized. Everyone may experience stress while being seriously ill, treated medically, or separated from loved ones in the hospital [170]. L-C19 can cause stress and uncertainty, affecting family dynamics. Caregiving and managing symptoms may require much attention from parents, as they may be emotionally unavailable or force the family to be less cohesive. Parents’ concerns about their children’s health may also stress their children. Biological mechanisms that may cause COVID 19 may lead to mental health problems. Infections with the COVID–19 virus affect the immune system. However, COVID-19–induced immune response may affect the brain, leading to mood disorders or cognitive problems. Anxiety and depression in children with L-C19 may result from brain inflammation or modifications in neurotransmitter activity [206,207,209]. While research is ongoing, it’s plausible that COVID-19’s physiological impact on the nervous and endocrine systems leaves children more vulnerable to mental health issues. For instance, inflammation might disrupt the balance of serotonin or other mood-regulating chemicals [205].

An important limitation of the current literature on mental health outcomes in pediatric long COVID-19 is the difficulty in disentangling the psychological effects of SARS-CoV-2 infection from the broader consequences of the COVID-19 pandemic itself. Prolonged social isolation, school closures, disruptions to daily routines, reduced access to peer support, and increased family stress have all been shown to adversely affect children’s and adolescents’ mental health, even in those without documented infection. Many published studies lack appropriate control groups of non-infected children exposed to the same pandemic-related stressors, which limits the ability to attribute anxiety, depression, sleep disturbances, and cognitive or behavioral changes specifically to long COVID-19 rather than to contextual factors associated with the pandemic [19,195,205]. Future studies should therefore incorporate well-matched control populations and longitudinal designs to better differentiate between infection-related neuropsychiatric effects and the indirect psychosocial impact of the pandemic environment.

Diagnosing a mental health condition in children with L-C19 is based on the symptoms, history, and emotional health of the child. It is necessary to have a detailed assessment of the child’s emotional state, behavior, and family history. Examples might include conducting interviews with the child and parents to determine the child’s mental health before and after COVID-19 infection. The mental health status of a child is assessed using standardized tools, such as questionnaires or assessments for anxiety, depression, PTSD, etc [210,211]. This fact enables their use to diagnose specific conditions and understand the severity of symptoms. In addition, it is important to screen for other conditions, such as ADHD or OCD, which may coexist or worsen with L-C19 symptoms. A comprehensive evaluation allows us to differentiate primary mental health disorders from those that might be set off or worsened by the pandemic [212]. Teachers, counsellors, and school psychologists can offer a lot to teach us about a child’s emotional and behavioral challenges. School reports help diagnose and manage mental health problems through their reports on attendance, performance, and social engagement.

#### 5.6.1. Management of mental health conditions in L-C19

CBT is one of the most effective psychotherapies for anxiety and depression [213]. CBT enables children to recognize and control negative thinking patterns and develop coping [214]. Family therapy is a way to address family dynamics and provide children with emotional support from their families. In rare cases, children need to prescribe medications, such as antidepressants or anti‐anxiety, under the guidance of a pediatric psychiatrist. If sleep disturbances play a role in mental health issues, medications that help sleep problems may be considered [156]. Social interaction still discourages children; however, getting them to do so, even if needed, may reduce isolation. Child support groups for L-C19 can give children a sense of community and normalcy. Children can find comfort in knowing they are not alone by connecting in peer support groups with people who understand their struggles [189]. Children with L-C19 can also get accommodations in school, perhaps modified schoolwork, more time to finish assignments, or more flexible attendance policies [190,215–217]. Reducing anxiety about school performance can be achieved by ensuring that children feel supported academically [156]. Practices of mindfulness, meditating, or performing breathing exercises can help children with anxiety and emotional regulation [33,190, 215,216].

### 5.7. Gastrointestinal manifestations

L-C19 causes several gastrointestinal (GI) manifestations in children, which are generally not as frequently discussed as respiratory or neurological issues [218]. This can take weeks or months and make a child’s life far more difficult [219]. In children, the GI symptoms of L-C19 can vary tremendously in severity and duration and may occur as part of a constellation of symptoms [218,219]. Abdominal pain is one of the most frequent GI symptoms in children with L-C19 is abdominal pain [51,104,218]. This can range from mild discomfort to very severe cramp-like pain, which usually occurs in the lower abdomen. The pain is often intermittent but may be constant and can be worsened by eating or drinking. It can interfere with a child’s ability to participate in activities that many children take for granted, such as school and sports [218]. Some children with L-C19 have persistent diarrhea, characterized by frequent loose stools [219]. It can occur a few times in a day and can occur alongside other GI symptoms, such as bloating and nausea. Diarrhea leads to dehydration, fatigue, and weight loss, which complicate recovery from L-C19 and worsen other symptoms [220]. GI complaints in L-C19 included nausea with or without vomiting. Although mild or severe, these symptoms can be triggered by food, activity, or even arise spontaneously. Nausea of longer durations can prevent children from having an appetite, which can prevent proper nutrition and hydration [219,221]. Many children with L-C19 also have a loss of appetite, which is usually associated with nausea, abdominal pain, or a lack of energy. Loss of appetite can also lead to weight loss, malnutrition, and reduced ability to recover. It may also impact the child’s overall energy levels, left feeling fatigued, and not being well able to do the day-to-day things that they want to [104]. Some children also complained of feeling bloated or had gas problems [222]. These symptoms can be related to changes in gut motility, alterations in gut microbiota, or inflammation of the gastrointestinal system following the viral infection. In kids with L-C19, constipation is less common [223]. Symptoms may be related to changes in diet, reduced physical activity, or the effect of other L-C19 symptoms such as fatigue and reduced appetite. Children have reported changes in the appearance of their stool, such as loose, watery stools, or fat stool, all signs of malabsorption or GI dysfunction [224,225]. Children with L-C19 sometimes develop new symptoms or have worse symptoms of gastroesophageal reflux disease (GERD), heartburn, acid regurgitation, and discomfort in the chest or upper abdomen. GERD has potentially been associated with inflammation in the stomach or esophagus accompanied by persistent pain after eating [222]. Some children with L-C19 may also have fewer common liver side effects, though very mild, such as elevated liver enzyme levels [181]. This may manifest as fatigue, abdominal upset, or changes in appetite. This could be due to either viral destruction of liver cells or damage to liver function by the immune system [226].

SARS-CoV-2 can enter the GI system and gain entry into the cells by binding to the ACE2 receptors, which are found in the lining of the gut, liver, pancreas, and other digestive organs [227]. Therefore, this could result in inflammation and dysregulation of normal gut function, particularly the virome [210,211]. Symptoms such as abdominal pain, nausea, diarrhea, and bloating can be caused by direct damage to GI cells by viral particles [211,212]. SARS-CoV-2 can cause widespread inflammation by forming an immune response throughout the body, including the GI system, and inflammation cascades can lead to symptoms such as diarrhea, abdominal discomfort, and altered gut function [212]. An overreaction of the immune system (inflammation) may also damage the lining of the gut, leading to more chronic symptoms [221]. It is well known that SARS Cov-2 can alter the gut microbiota and community of bacteria or other microorganisms present in the intestines [228–232]. An imbalance in these microbes can upset normal digestion and cause symptoms such as diarrhea, constipation, and bloating when the virus and the inflammatory response are prompted [229]. An imbalance of gut bacteria (dysbiosis), which can last for a long time, is associated with conditions such as irritable bowel syndrome (IBS). Persistent GI symptoms in children with L-C19 could be another manifestation of dysbiosis [210,221,229]. Problems in the ANS can also cause gut movement and digestion to slow or lead to problems such as bloating, nausea, constipation, or diarrhea [211,230]. This is especially important in children with L-C19 and other symptoms such as dizziness or fatigue, suggesting a more general impairment of autonomic function [199]. Whatever stress and psychological burden accompany a long-term illness, such as L-C19 can also impact gut health [231,232]. Stress may change the gut-brain axis and worsen nausea, constipation, and diarrhea [233,234]. Changes in diet, decreased physical activity, and the emotional toll of managing long-term illnesses can compounding issues that negatively impact gastrointestinal function. Some treatments for L-C19 symptoms in children can also cause gastrointestinal symptoms [210,226].

GI symptoms in children with L-C19 are diagnosed through clinical evaluation, symptom history, and exclusion of other causes. Many children with L-C19 may have elevated liver enzymes, markers of systemic inflammation, or electrolyte imbalances [226,235]. Additional stool tests can be performed to identify infectious processes or parasites that could explain gastrointestinal symptoms, but these are typically negative for L-C19 GI issues. In the case of severe symptoms, abdominal imaging (ultrasound or CT scan) may also be used to exclude other GI pathologies (such as appendicitis, gallbladder disease, or inflammatory bowel disease). If symptoms persist and there is suspicion of more significant GI pathology (for example ulcers or inflammation), endoscopy may be needed, such as an upper GI endoscopy or colonoscopy, to examine the lining of the stomach or intestines. If dysbiosis or motility issues behind the symptoms are suspected, tests such as breath testing for bacterial overgrowth or motility may be ordered [210–212,227–235].

#### 5.7.1. Management of gastrointestinal symptoms in L-C19

For the management of GI symptoms in children with L-C19, symptom relief, nutritional support, and treatment of any underlying cause have been emphasized [236]. For persistent diarrhea, antidiarrheal drugs such as loperamide may be used to reduce nausea and vomiting. Laxatives and fiber supplements may be prescribed for constipation [210]. The diet may be rich in nutrients, but also low in fat and cholesterol, and may be bland, low-fiber foods initially and then progressed to a more normal diet as symptoms improved [237]. Lost fluids and electrolytes are usually restored using oral rehydration solutions. If there are signs of dysbiosis, probiotics may be considered to add in order to help restore a healthy balance of gut bacteria, promote protective antiviral responses, and inhibit excessive inflammatory responses of the host [235,238]. Stress and anxiety worsen GI symptoms and need to be addressed as part of L-C19. Stress can be managed using CBT or relaxation techniques, and GI symptoms can improve [239]. Light physical activity should be encouraged to stimulate bowel function, reduce stress, and improve overall GI motility [240].

### 5.8. Dermatological manifestations

Less commonly discussed, dermatological manifestations in children with L-C19 can play a substantial role in the overall presentation. These skin-related symptoms can be mild to severe and continue after the acute stage of COVID-19 [241]. One of the most common dermatological symptoms in children with L-C19 is rash. These rashes may occur several weeks after infection and differ in appearance, location, and severity. Some of the specific types of rashes observed in L-C19 include maculopapular rash, urticaria, viral exanthema, and morbilliform rash [242]. Some children with L-C19 have noticed a condition called COVID toes or pernios. Redness, swelling, and sometimes blistering of the toes or fingers characterize this condition. Symptoms of COVID in toes generally develop approximately a few weeks after the initial infection and may be painful or itchy. This may be due to the inflammation of small blood vessels (vascular inflammation), resulting in reduced blood flow to the extremities, causing swelling and discoloration of the area [243]. Some children with L-C19 have reported alopecia or hair loss [244]. The most common type of hair loss is termed *Telogen effluvium*, in which hair progresses prematurely into the resting phase and falls out in greater amounts than normal. Unlike other types of hair loss, this typically occurs several weeks to months after becoming sick and, while it can be distressing, fortunately, is usually temporary [245]. After viral infection and inflammation settle, hair often grows back. Rarely, some children with L-C19 have dry skin or skin peeling, especially on their hands and feet [246]. This may be due to the activation of the immune system and skin inflammation. Dry skin can be uncomfortable, with cracks or flakes, and may need moisturizing and the use of gentle skincare products to prevent irritation. There is a documentation of children with L-C19 presenting with new psoriasis-like lesions [241]. Some children may have a flare-up of previous psoriasis, whereas others may develop a rash resembling psoriasis. These lesions can be painful and require treatment with topical steroids or other inflammatory drugs. A viral exanthema or rash that develops as a consequence of a viral infection may appear in a child after recovery from the acute phase of COVID-19. These rashes can be of many types, including maculopapular rashes and vesicular eruptions, and may be due to an immune response to the virus [247]. *Livedo reticularis* is a condition that causes discoloration of the skin in a mottled, purplish, lace-like pattern. It is caused by blood flow issues or inflammation of the blood vessels underneath the skin [241,248]. This condition has been observed in children and adults with L-C19, and although it is sometimes temporary, when it occurs, it can be concerning because it could mean underlying vascular changes or immune responses. Sometimes, a rash can be a symptom of systemic inflammation, such as the immune system overreacting and creating widespread inflammation that causes changes in the skin. They might also include a rash that appears with other L-C19 symptoms such as fatigue, muscle aches, or neurological symptoms. The rash may be diffuse, reddish, and have multiple body areas [246,248].

While the direct viral effects of SARS-CoV-2 on the skin are still being investigated, it is believed that the virus may infect skin cells or interact with part of the immune system to induce skin changes [249]. ACE2 receptors are expressed in the skin and other tissues, and SARS-CoV-2 is believed to enter skin cells through these receptors [250]. An overreaction of the immune system to the virus can result in a cytokine storm: the release of excessively high amounts of immune proteins called cytokines that trigger widespread inflammation [242,251]. Such presentations may lead to different types of dermatological manifestations, such as rashes and pernio (COVID toes). COVID toes or *livedo reticularis* are conditions that develop because of inflammation caused by COVID-19 and affect small blood vessels (microvasculature) in the skin [249]. Blood flow abnormalities in areas of increased blood flow are associated with these skin changes and may be caused by endothelial damage from the viral effect on the vascular system [246]. The body’s reaction to a viral infection may result in some dermatological symptoms. Even after the virus has cleared from the body, it may continue to activate the immune system, causing skin rashes or flareups of pre-existing skin conditions such as psoriasis [241]. It can also flare up or trigger skin conditions that can be emotionally and psychologically stressful. Dermatological manifestations in affected children may be caused by flareups of eczema, psoriasis, or hives due to stress [252]. Signs and symptoms that some children with L-C19 might receive medications (e.g. antivirals, antibiotics, anti-inflammatory drugs) can have side effects (e.g. rashes). Drug-induced rash may mimic or contribute to other dermatological symptoms [253,254].

The diagnosis of dermatological manifestations in children with L-C19 requires a detailed history of rash onset, progression, and associated symptoms. This differentiates between the dermatological symptoms of L-C19 and those of other rashes. In other cases, the skin was biopsied and examined under a microscope. Blood tests for highlighting the signs of systemic inflammation, immune activation, or underlying infections may help confirm the underlying cause of the rash, such as viral exanthema, or to check whether the rash is related to systemic inflammation. In addition, potential inflammatory components or specific antibodies may be elevated. Patch testing can be helpful in determining whether a drug or other substance triggers a rash. However, if dermatological symptoms are poorly controlled, unusual, or severe, referral to a pediatric dermatologist is warranted for further evaluation and management.

#### 5.8.1. Management of dermatological symptoms in L-C19

The treatment of dermatological manifestations of L-C19 in children involves symptomatic treatment and treatment of the underlying condition [255,256]. Rashes and psoriasis-like lesions can be treated with corticosteroid creams or ointments to reduce inflammation and itching, and emollients and moisturizers are advised for dry skin or peeling [257]. They can also restore the skin barrier function and decrease discomfort. Urticaria (hives) or rashes with itching may be treated with oral or topically [250,251]. In the case of COVID toes, comfortable shoes and trying not to wear tight footwear can help to relieve discomfort, and in severe cases, topical corticosteroids may be used [243]. Children with hair loss (*telogenic effluvium*) should follow gentle hair-care practices. Most of the time, hair loss is temporary, and hair regrows as the body recovers from the virus [39]. If the rash is very bad or covers a large area of the body, oral corticosteroids or immunosuppressive medications can be recommended to help control inflammation [251]. However, general supportive measures such as staying hydrated, eating a balanced diet, and dealing with stress can also help improve a child’s skin health and overall wellbeing [254].

### 5.9. Musculoskeletal manifestations

Musculoskeletal manifestations are an important and often distressing part of L-C19 in children. Musculoskeletal pain, including joint pain, muscle aches, and fatigue, can cause children to participate in daily activities, such as school, sports, and recreational activities [1343]. Joint pain (arthralgia) is one of the most commonly reported musculoskeletal symptoms in children with L-C19. It may be intermittent or persistent, and may involve one or multiple joints [32,139]. The pain can be mild to moderate and may affect the knees, wrists, ankles, and elbows. The pain may worsen after physical activity, making it difficult for children to engage in normal activities or exercise. Another common complaint is muscle pain (myalgia), which can occur throughout the body or in certain areas [139,141,258]. Without strenuous activity, children might feel as if their muscles are sore or tender. Localized (restricted to one area, such as the back, shoulders, or legs) or generalized joint pain may occur. Muscle aches may be enhanced in the morning or after activity, and may continue for several months or even years after the acute stage [258]. Fatigue is one of the most common L-C19 symptoms, and is usually associated with severe musculoskeletal discomfort. Even children can be so fatigued that they cannot be properly restored, hindering their ability to attend school, play, and perform other daily living routines [31,35, 136,259]. Fatigue is sometimes accompanied by muscle weakness and worsening of symptoms after exercise or other physical activities (post-exertional malaise). This condition is related to myalgic encephalomyelitis/chronic fatigue syndrome (ME/CFS), a multisystemic disease characterized by fatigue and musculoskeletal pain [137]. Another musculoskeletal problem in some children with L-C19 is muscle weakness. Consequently, this can create problems with activities such as walking upstairs, carrying objects, or playing sports. When general fatigue involves large muscle groups, such as the legs or arms, it can affect a person’s ability to move and coordinate movement [38]. Some children with L-C19 develop joint swelling or inflammation, which, although less common, can resemble inflammatory arthritis. The joint(s) may be swollen, tender, or stiff, and children may be unable to move the affected joint or may stop using the joint in an attempt to avoid pain [260,261]. Another musculoskeletal symptom of L-C19 infection in children is back pain. This may be due to muscle strain, poor posture due to fatigue, or prolonged bed rest during the acute phase of COVID-19. The causes of back pain can be mild to severe and may result in muscle stiffness or spasms. In some cases, it may also cause difficulty in maintaining a regular routine because it can be performed while sitting or standing for long periods [258]. Pain in tendons or ligaments may present as tendonitis in some children. The main reason for this pain is the inflammation of the tendons around the joints, primarily in the elbow, shoulders, and knee. Joint stiffness and restricted mobility are the consequences of tendonitis. Post-exertional malaise (PEM) is an increase in musculoskeletal pain, fatigue, or other symptoms after physical or mental effort [146]. With L-C19, children may feel much worse after physical or even mental stress, with symptoms such as muscle aches, joint pain, and worsening fatigue. PEM can then make it difficult for children to return to activities such as school or sports without seeing a flare-up of symptoms that can last for days or even weeks [147].

COVID 19 flips the switch on the immune system, causing it to react as if it encounters a pathogen, triggering inflammation throughout the body [262]. This causes the immune system to become ‘dysregulated’ which can lead to chronic muscle and joint inflammation, resulting in chronic muscle pain, chronic joint pain, and chronic muscle weakness. In other instances, the immune response can be too strong, and an autoimmune-like reaction can occur, where the body attacks its own tissues, leading to pain and damage to the muscles and joints [263]. SARS-CoV-2, the virus responsible for COVID-19, may directly affect musculoskeletal tissues. The virus is mainly known to invade the respiratory system, but it has also been found to damage other organs and systems, such as the muscles and joints [264]. The virus may cause direct injury to muscle fibers, leading to muscle pain and weakness. It might also impact the microvascular system, causing inadequate blood flow to muscles and joints, and thus causing inflammation and pain. LC-19 shares many similarities with myalgic encephalomyelitis/chronic fatigue syndrome (ME/CFS), a condition that can develop after a viral infection [101,265,266]. The fatigue, muscle pain, and joint pain in ME/CFS are alike but still similar to those seen in L-C19 and result from abnormal response of the body to the virus, which continues after the acute sickness has settled [266]. It can consist of post-exertional malaise, which can exacerbate musculoskeletal symptoms following physical activity, and general weakness, fatigue, and pain in the muscles and joints. In some people, COVID-19 can cause nerve damage that can result in pain, muscle weakness, and altered limb sensations. The virus can cause inflammation of the spinal cord or nerves that control muscle movement (which leads to musculoskeletal symptoms such as pain or weakness). Poor coordination and balance can develop as a result of neurological issues and can cause musculoskeletal complaints [101].

Diagnosis of musculoskeletal manifestations in children with L-C19 includes a history of symptoms, such as onset, site, and manner of pain or weakness, and changes in physical activity or fitness during the illness. Blood tests may be conducted to check for signs of inflammation, such as CRP or ESR. These markers can indicate whether there is ongoing inflammation or immune system activity that might be contributing to musculoskeletal symptoms. Tests for certain autoimmune markers may also be ordered if an autoimmune cause of the symptoms is suspected [267]. Occasionally, X-rays, MRI scans, or ultrasound can be used to exclude structural abnormalities or damage to a joint or muscle. Imaging can detect signs of joint inflammation or soft tissue damage; however, in L-C19, musculoskeletal pain imaging is often normal [50]. A functional assessment may be performed to determine how well the child moves (range of motion), how much strength he or she has, and overall, how well the child functions physically. It may also help gauge the effects of musculoskeletal symptoms on children to perform normal daily activities [265].

#### 5.9.1. Management of musculoskeletal symptoms in L-C19

Joint and muscle pain may be treated with over-the-counter pain relievers, such as acetaminophen or ibuprofen. Other types of pain relievers, such as creams or gels that are applied to the skin, can also be used in the treatment of sore muscles and joints. Children who need back muscle strength, flexibility, or function often undergo physical therapy [50,212]. Physical therapists can create an exercise plan to overcome muscle weakness, joint pain, and increased mobility. Stiffness and endurance can be improved by stretching and strengthening exercises, and young patients who experience post-exertional malaise should be encouraged to return to physical activity gradually [212,268–271]. Low-intensity activities are best started with and drawn up gradually to prevent overexertion and further deterioration of symptoms. Initially, children should not push themselves too hard to avoid exhaustion or worsening musculoskeletal symptoms. Children should balance physical activity with enough rest to help their body heal and avoid PEM [271]. As stress and anxiety worsen physical symptoms, attending to mental health is an important part of recovery. Children with such symptoms recover with a combination of symptom management, physical therapy, and a gradual return to activity; however, children with persistent or particularly severe musculoskeletal issues may need ongoing support and monitoring [270,271].

### 5.10. Hematologic presentations

Compared to other systems, hematologic manifestations in children with L-C19 are less frequently discussed but are nonetheless important. These manifestations may include abnormalities in blood cell counts, clotting, and coagulation pathways, and can reflect the ongoing effects of SARS-CoV-2 on the body’s immune and vascular systems [272]. L-C19 hematologic issues in children include a spectrum of mild and transient changes and more serious complications, such as inflammatory or autoimmune responses. Children with L-C19 may experience a low platelet count, known as thrombocytopenia [273]. Thrombocytopenia occurring in L-C19 could be due to the destruction of platelets by the immune system, or it could be caused by secondary inflammation or infection with a virus. It often moves away as the child recovers from the viral infection [274,275]. Thrombosis, or the formation of abnormal blood clots, is one of the more serious hematologic concerns associated with L-C19, although it is less common in children than in adults. They develop in different parts of the body, such as the veins (venous thrombosis) or arteries (arterial thrombosis). Hypercoagulability has been associated with L-C19 and is linked to an increased risk of clotting. It is thought that the mechanisms underlying this increased risk of clot formation are related to endothelial damage, immune activation, and a pro-inflammatory state. This can cause several complications, including deep vein thrombosis (DVT), pulmonary embolism, and stroke [276].

Elevated D-dimer levels represent increased clot formation and breakdown in the body, and it is commonly seen in children with long COVID, especially in those who have a history of acute infection or thrombotic events. An elevated D-dimer level may signal the presence of microclots in the body, a known issue in LC-19, and can be a clue to clinicians that a patient may be at risk of clotting complications [80,276]. Some children with LC-19 may develop mild to moderate anemia, often related to ongoing inflammation, nutritional deficiencies, or the body’s response to viral infection [273]. Fatigue, weakness, and poor exercise tolerance are extremely difficult to overcome in cases of anemia; therefore, it is harder for children to return to normal activities. L-C19 can even cause leukopenia or a low white blood cell count. Leukopenia can develop during the acute phase of COVID-19, but it can later become chronic during the L-C19 phase owing to immune dysregulation or through the action of the virus on bone marrow function [277]. Another hematologic manifestation in children with L-C19 is lymphocytosis. In addition, this could be a sign of chronic immune activation; the body is still sending the signal, ‘there is a virus here and we need to keep going’. White blood cell—lymphocyte ratio increases in the body when there is inflammation or immune system activity [277,278]. Children with L-C19 may also have coagulation abnormalities owing to disruptions in the normal clotting process [279]. Clinically, this can present, for instance, prolonged clotting times and abnormal clotting factor levels, and can predispose to bleeding and thrombosis. Coagulation abnormalities are believed to result from persistent inflammation or a continuous response of the coagulation pathway to viruses. SARS‑CoV‑2 can cause inflammation of the endothelium, resulting in disrupted blood flow that induces clots or thrombosis. In L-C19, endothelial dysfunction can continue to increase the risk of microvascular clotting and other blood issues. During infection, inflammatory cytokines can stimulate clotting factors; in addition to the hypercoagulable state, this results in thrombosis [280]. Hematological manifestations of L-C19 are mediated by immune system dysregulation. Following the first infection, the immune system may become too active, continuing to produce inflammatory markers and antibodies that can interfere with blood cells. In addition to hematologic problems, autoimmune reactions may contribute to conditions such as thrombocytopenia, the destruction of platelets by the immune system, or the emergence of autoimmune anemia, since the bone marrow can no longer produce normal blood cells in the chronic inflammatory state seen in L-C19 [281]. Chronic inflammation may also disrupt the production of red blood cells (anemia) or white blood cells (leukopenia or lymphocytosis). In addition, inflammation that continues over time can turn over coagulation pathways, leading to clotting disorders or extended clotting times [282]. In some situations, bone marrow suppression can occur, resulting in lower counts of one or more blood cell types. Anemia or leukopenia in children with L-C19 may have been caused by this. Disruption of blood cell production in the bone marrow secondary to viral infection and immune-mediated damage may result in multiple hematologic abnormalities [283,284]. Some hematologic issues may be explained by autoimmune responses, whereby the body’s own immune system produces antibodies that attack cells, including blood cells. It becomes especially relevant under conditions of immune thrombocytopenic purpura (ITP), where the immune system attacks platelets [283].

Hematological issues were assessed using complete blood count (CBC). A CBC can help pick up thrombocytopenia, anemia, leukopenia, or lymphocytosis [285]. One way to test for abnormal clotting and microclots in the bloodstream is by measuring D-dimer levels, which reflect ongoing clotting or breakdown. Tests to check for clotting or bleeding may include blood tests in a lab called a coagulation profile that may include prothrombin time (PT) or activated partial thromboplastin time (aPTT) [286]. Special antibody tests or autoimmune panels may be performed in the presence of suspected autoimmune-related blood cell destruction (such as thrombocytopenia or hemolytic anemia) [278]. In extremely rare situations with severe or persistent hematologic abnormalities, bone marrow biopsy may be performed to rule out bone marrow suppression or malignancy [287]. When thrombosis is suspected (for example, deep vein thrombosis or pulmonary embolism), imaging techniques such as ultrasound or CT pulmonary angiography may be performed to identify blood clots.

#### 5.10.1. Management of hematologic manifestations in L-C19

Children with L-C19 who develop hematologic manifestations require regular monitoring of blood counts and coagulation parameters. In severe cases, blood transfusions or injections of platelets may be warranted in cases of anemia or thrombocytopenia [282]. Anticoagulant therapy (e.g. heparin, low-molecular-weight heparin) may be indicated for children with evidence of thrombosis or other risk factors for the development of blood clots to prevent further clot formation [286–288]. The clinical presentation, in combination with the risk factors, will lead to the decision to begin anticoagulation therapy [287]. Steroid and immunosuppressive treatments may be used to suppress the immune response if a blood cell disorder is thought to be caused by the immune system (e.g. immune thrombocytopenic purpura or hemolytic anemia) [289].

## 6. Relationship between long COVID and multisystem inflammatory syndrome in children (MIS-C)

Multisystem inflammatory syndrome in children (MIS-C) and long COVID are different post-SARS-CoV-2 outcomes in children, which differ significantly in terms of timing, clinical expression, pathophysiology, and prognosis, although both are events that follow acute infection [290]. MIS-C is an acute and delayed hyperinflammatory disease that usually occurs 2–6 weeks after a SARS-CoV-2 infection and commonly occurs after an asymptomatic or mild acute infection [291]. LC-19 (post-COVID-19 condition), on the other hand, is a condition characterized by the introduction or continuation of symptoms weeks to months after infection, usually 12 weeks and longer, and with a chronic or fluctuating pattern as opposed to an acute one [292].

The MIS-C is characterized by a high fever, severe systemic inflammation and involvement of multiple systems, most commonly cardiovascular, gastrointestinal, mucocutaneous, and hematologic systems. Recurring conditions include cardiac dysfunction, shock and intensive care [293–296]. In children, LC-19, on the contrary, is predominated by non-specific and persistent symptoms, including fatigue and exercise intolerance, cognitive impairments, sleep problems, headaches, and mood disturbances [11,19]. The functional involvement of the organs in LC-19 is typically not structural but functional and life threat manifestations are rare [41]. While L-C19 can present with prolonged symptoms such as fatigue, cough, and brain fog, MIS-C is far more severe and characterized by systemic inflammation and multiple organ dysfunction [293]. The symptoms of MISC vary as it is a systemic inflammatory response [80,275,276]. Clinical features are common and include persistent high fever (>24 h), which is more or less unresponsive to typical fever reducing medications [294]. MIS-C is associated with abdominal pain, vomiting, diarrhea, and nausea, sometimes due to gastrointestinal inflammation [212,267]. In children, myocarditis or pericarditis is a serious complication; children may present with tachycardia, hypotension, and shock, which are life-threatening [297–299]. In very severe cases of MIS-C, coronary artery dilation or aneurysms or even long-term heart problems with proper treatment have been reported [297,298]. Although not as common as in adults, the lungs may also be damaged, and children with MIS-C may have respiratory distress or pneumonia, although often it is because the inflammation has spread to the lungs [300]. Common symptoms are a characteristic rash (erythematous or similar to that of Kawasaki disease) and conjunctivitis (red eyes) [301]. The rash can be similar to scarlet fever or other viral rashes, and conjunctivitis is almost always nonpurulent. Neurological symptoms, including headache, irritability, lethargy, or dizziness, can occur in children. In rare cases, seizures, confusion, or disorientation may occur [302]. A common feature is swelling of the hands and feet, often with redness or tenderness that reflects inflammation of small blood vessels [303].

It is believed that MIS-C is caused by a post-infectious immune dysregulation, which is excessive release of cytokines, activation of T-cells, and production of autoantibodies, which cause acute organ damage [283–286]. Suggested mechanisms are the activation of immune through superantigen and abnormal humoral responses [287]. LC-19 seems to be heterogeneous in pathophysiology, which is included in persistent viral antigens, chronic low-grade inflammation, autoimmunity, endothelial dysfunction, dysautonomia, and poor tissue recovery. LC-19 does not have one general inflammatory signature, as MIS-C does [51,52]. Recent definitions provided by the World Health Organization and other sources do not consider MIS-C a subset of LC-19 since MIS-C is a well-recognized time-limited syndrome whose definition includes definite diagnostic criteria [291]. LC-19, in turn, is an exclusion diagnosis that is used when the symptoms continue to be present and there is no other reason [4,15]. However, a proportion of children who have recovered MIS-C can have transient post-acute symptoms including fatigue or decreased exercise capacity, and go over with LC-19 symptoms. Follow-up studies that have been available show that in the majority of MIS-C cases, most survivors recover completely in 6–12 months, and there are also low rates of long-term organ injuries, which argues that MIS-C does not commonly result in long-term LC-19 [302,304–306]. To conclude, MIS-C and LC-19 are some of the biologically and clinically different post-COVID conditions in children. MIS-C is an acute, immune-mediated, multisystem inflammatory syndrome occurring shortly after infection, whereas LC-19 is a chronic and heterogeneous condition with persistent symptoms and functional deficiency. These entities should be clearly differentiated in order to diagnose, manage, and prognostically counsel them.

## 7. Markers of L-C19 in children

Despite several definitions for L-C19 in children, laboratory criteria that may be used as markers are still under study [4]. Typical tests such as D-dimer, C-reactive protein, antinuclear antibody, and complete blood count are often normal in this category of patients [307]. A study performed in 2024 by Kovács et al. on 129 children with L-C19 syndrome (LCS) concluded that there is no significant difference in Interferon Gamma (IFN-γ) levels in the plasma of long COVID syndrome patients compared to the control group, while interleukin 6 (IL-6) concentration is higher [308]. Espin et al. suggested the use of IFN-γ and IL-6 as biomarkers of L-C19 syndrome [212]. The cell dysfunction that appears in LCS can be a possible explanation for virus reactivation (Epstein Barr, Human herpes Virus 6), as reported by Davis et al. [309], and streptococcal tonsillitis, as reported by Mizrahi et al. [310]. The study of Gűven and Buluș conducted on 251 children in a tertiary single center hospital in Ankara between August and October 2021 concluded that increased leukocytes, monocytes, neutrophils, basophiles, platelets and D-dimer were statistically significantly higher in patients with L-C19 compared to those without LCS [20]. Camporesi et al. conducted a study on 1319 patients in a pediatric post-COVID clinic in Rome, initially, out of whom 1296 reached the 3 months follow up, and then underwent multiple follow-up examinations at 3-6-12-18-24 months, and found a high proportion of new diagnoses (1.1%–15 cases) of autoimmune diseases following SARS-CoV-2 infection in children [311]. The 1.1% prevalence is higher than that reported in other studies, which is approximately 0.4% [312,313].

## 8. Long COVID and SARS-CoV-2 viral variants

According to new evidence, the risk and clinical manifestation of LC-19 can depend on the dominant type of SARS-CoV-2 variant, but the data on children are insufficient and heterogeneous [314]. Initial reports of the ancestral strain and Alpha variant periods tended to describe more persistent symptoms, which probably covered both pathogenicity of the virus and lack of population immunity [315]. Subsequent waves with Delta predominant have been linked to more severe acute disease in certain pediatric groups and hence, concerns of more long-term sequelae. However, results of prevalence of LC-19 following a Delta infection have been unstable. The burden of lingering symptoms was found to relate to Delta infections in children as compared to previous strains. As an illustration, Italian cohort reported that children infected in the Delta wave had more frequent reports of fatigue, weakness, and other post-COVID complaints compared to those infected in the previous Alpha/wild-type period [316]. In contrast, the Omicron wave (since late 2021) experienced a viral increase in cases among children, but apparently, a reduced percentage of LC-19 cases per infection [317]. Several studies sustain the fact that the risk of LC-19 in the young population has been declining with the each passing wave of variants. A pediatric cohort in Thailand found overall long COVID prevalence of ∼30%, with the Delta period having a significantly higher rate than the Omicron period (36.3% vs 23.9%) [318]. On the same note, a study by Turks comparing 200 children (Delta vs Omicron era) reported that post-COVID fatigue, weakness, exercise intolerance, anxiety, and gastrointestinal disturbances were significantly more prevalent after Delta infections compared to Omicron infections [314]. In that study, prolonged fatigue affected 22.5% of Delta-infected children versus 8.5% of those infected during Omicron, and other symptoms like chronic anxiety were almost exclusive to the Delta group [314]. These data indicate that Omicron infections are more likely to result in persistent symptoms in a smaller proportion of pediatric cases and the profile was milder than Delta. It is noteworthy that an in-depth study in the United Kingdom did not identify any significant difference between the symptom profiles and impacts of 12 months in both children infected during the Omicron wave and during Alpha/wild-type waves [318]. It implies that even though the prevalence of pediatric long COVID may have decreased with Omicron, the syndrome itself was generally similar. Positively, the majority of children with all variants had recovered without any severe long-term complications. Researchers are exploring the possible occurrence of new long COVID patterns in children with Omicron sublineages (e.g. BA.2, BA.4/5 in 2022 and more recently XBB.1.5, EG.5, BA.2.86 in 2023). So far, no specific variant-related differences in pediatric long COVID were observed to be associated with these subvariants. Initial data points to the risk of long COVID being the same across Omicron sub-lineages. An example is a large cohort study of COVID survivors (the majority adults, but including Omicron BA.1 to XBB subvariants) which determined that risk of long COVID was equal between Omicron BA.1 and BA.XBB infections [318]. Pediatric-specific data on variants such as EG.5 (‘Eris’) or the highly mutated BA.2.86 (‘Pirola’) are still limited, but so far these variants have not been associated with any unique long COVID symptoms or increased prevalence in children. The important confounders in the interpretation of variant-specific effects include rising levels of hybrid immunity, alterations in testing, development in clinical awareness, and study design differences. Furthermore, there is a general similarity of symptom profiles in variants, with fatigue, cognitive problems, and exercise intolerance being the most frequently reported. Longitudinal, variant-stratified studies with the proper combination of control groups are necessary to understand whether pronounced pathophysiological processes are the cause of long COVID after the different SARS-CoV-2 variants in children and adolescents.

## 9. Conclusions

L-C19 has emerged as a clinically relevant post-infectious condition in children and adolescents, characterized by heterogeneous, multisystem manifestations that may persist for months after acute SARS-CoV-2 infection. Although the overall prevalence of pediatric long COVID appears lower than in adults and has declined in more recent variant waves, a meaningful subset of affected children experience symptoms that interfere with physical functioning, cognitive performance, mental health, and quality of life. Notably, even mild or asymptomatic infection may result in long COVID, and it may be observed in children with no underlying conditions, which explains the necessity of general clinical awareness. Current evidence highlights several priorities to optimize care and improve long-term outcomes. First, early recognition and standardized assessment are essential. A high index of suspicion of long COVID in children with persistent, unexplained post-infection symptoms should be highly observed by clinicians, and with alternative diagnosis being systematically ruled out. Pediatric-specific clinical assessment instruments and outcome measures would be adopted, and this would enable more consistent diagnosis, follow-up, and cross-studies and clinical settings. Second, it is based on the available literature where a multidisciplinary, symptom-focused management approach is suggested based on the chief complaints and functional limitations dominating in the child. This may involve the use of coordinated care with pediatricians, rehabilitation specialists, cardiology, neurology, mental health professionals and educators especially in children who experience fatigue, autonomic symptoms, cognitive difficulties or school impairment. Rather than emphasizing on aggressive or uniform treatment protocols, pacing strategies, gradual return to activity, psychosocial support and family education should be emphasized. Third, high-quality longitudinal studies of pediatrics are urgently required. Future research should prioritize well-designed prospective cohorts with appropriate control groups to distinguish the direct effects of SARS-CoV-2 infection from the broader psychosocial consequences of the pandemic. Research is also needed to investigate the biological processes, to determine effective biomarkers, and uncover risk and protective factors, such as the functions of age, sex, vaccination, reinfection, and viral strains. This information is essential to optimize the diagnostic criteria, target the vital interventions, and detect the children at risk of a long and severe disease course. Lastly, to maximize the outcomes of children with L-C19, the healthcare system is not the only area that should be addressed. Accommodations of education, social support and public health interventions are part of reducing long-term developmental and psychosocial outcome. Such continuous monitoring and partnership of the clinicians, researchers, school, and policymakers will be necessary to make sure that the affected children are not forgotten after the acute stage of the pandemic has been left behind. To sum up, pediatric L-C19 represents a complex but increasingly understood condition. Further investment in standardized clinical pathways, interdisciplinary support patterns, and rigorous pediatric research will be key to improving recognition, management, and long-term outcomes for children and adolescents living with L-C19.

## Data Availability

No datasets were generated or analyzed during the current study.
